# Mathematical modeling and optimal control of depression dynamics influenced by saboteurs

**DOI:** 10.1038/s41598-025-90357-w

**Published:** 2025-02-25

**Authors:** S. Nivetha, A. Karthik, Abhinav Tandon, Mini Ghosh

**Affiliations:** 1 Department of Mathematics, School of Advanced Sciences, Vellore Institute of Technology, Chennai Campus, Chennai, Tamil Nadu 600 127 India; 2 Department of Mathematics, Birla Institute of Technology Mesra, Ranchi, Jharkhand 835215 India

**Keywords:** Psychology, Mathematics and computing

## Abstract

Depression disorder affects millions globally, characterized by symptoms such as profound sadness, loss of interest in activities, and disruptions in eating and sleeping patterns. Understanding depression within the context of chronic pain is essential for developing effective management and intervention strategies. This study utilizes mathematical modeling to analyze depression trends using empirical data from Spain spanning from 2011 to 2022. Our depression model incorporates distinct compartments for primary and secondary depressed populations, along with a category for individuals categorized as saboteurs, who may actively influence the depression prevalence. We calculated the basic reproduction number $(R_0)$ and identified four equilibrium points and evaluated their stability. Additionally, sensitivity analysis was conducted to assess the impact of $(R_0)$ on depression prevalence. Furthermore, optimal control strategies were explored for the model. These strategies aim to improve treatment adherence, encourage doctor consultations, promote self-medication practices, and enhance recovery rates, ultimately aiming to reduce spread of depressive disorders and associated mortality. Data fitting was conducted using Python, and simulations were carried out in MATLAB to ensure rigorous validation of the model.

## Introduction

Millions of individuals worldwide suffer from depression, a common mental health problem that affects people of all ages, cultures, and backgrounds. It manifests in various forms and presents a significant public health challenge. Understanding the prevalence, causes, and consequences of depression is essential for addressing this issue. The causes of depression are diverse, including biological factors such as altered hippocampal function, inhibition of neurogenesis, and reduced BDNF levels^1^. Additionally, changes in glucocorticoids and CRH levels due to stress contribute to the development of depression^2^. Social and environmental factors also play a crucial role. Stressful family environments, interpersonal difficulties, and negative life events are significant contributors^3^. Financial hardship, especially among single parents, is associated with feelings of depression and anxiety^4^. Debt has been shown to strongly correlate with mental health issues, negatively affecting both individuals and families^5^. Since the 1970s, the prevalence of depression has risen, showing a strong association with physical health problems^6^. Individuals with depression often exhibit pessimistic prediction styles and low future expectations, contributing to the onset and persistence of depressive symptoms^7^. Studies have also demonstrated the key role that stress, neuroplasticity, and emotion regulation play in both the development and maintenance of depression^8–12^.

In recent years, mathematical modeling has emerged as a vital tool in analyzing the complex interactions of biological, psychological, and social factors contributing to depression. These models help predict outcomes of different treatment approaches, offering valuable insights into personalized treatment plans that consider patient subpopulations and antidepressant response patterns^13,14^. Previous studies^15–17^ have employed compartmental and mathematical models to investigate various aspects of depression. For instance, in^15^, authors formulated a compartmental model to analyze the interplay between social media addiction and depression, focusing on equilibrium points, reproduction number $R_0$, bifurcation behavior, and sensitivity analysis. While insightful, this work does not consider external ’saboteurs’ influencing the dynamics of depressive disorders. In^16^, authors derived a mathematical model for spreading depression based on ionic concentration dynamics, incorporating physiological processes like the conductance of ions $K^+$ and $Ca^{++}$, coupled with diffusion equations. However, this model remains limited to physiological mechanisms and does not explore population-level trends or intervention strategies. Similarly, authors in ^17^ presented a model of mood dynamics rooted in psychological theories of unipolar clinical depression, demonstrating how stress factors under certain conditions can trigger depressive episodes. Despite its utility for analyzing emotional states, this work does not account for external influences or interventions at the population scale. Another study presents a fractional model for managing online gaming addiction, providing insights into control strategies to mitigate its negative effects^18^. A model examining depression in young women highlights the influence of vulnerability, treatment, recovery, and peer pressure on depression progression^19^.

Optimal control theory is often integrated into mathematical models to identify the most cost effective strategies to achieve desired outcomes within a stipulated time frame. This approach provides valuable insights into decision-making processes by examining the factors and constraints in a given scenario. For example, optimal control has been used to address transmission dynamics in malaria, dengue, and COVID-19, applying Pontryagin’s Maximum Principle to derive time-dependent interventions aimed at reducing disease prevalence and economic burden^20–23^. In the context of depression, optimal control can help determine the best combination of therapy options, counseling frequency, and treatment duration to minimize symptoms and improve quality of life. One study used an inverse optimal control approach to reveal the relationship between depression severity and performance deficits, including altered sensorimotor speed and motivational dysfunction^24^.

Mathematical models have also been used to explore the spread of depression through social interactions. For instance, a nonlinear SIR model was applied to investigate the spread of depression, highlighting the importance of therapeutic interventions^25^. Building upon these insights, we developed a novel mathematical model of depressions and validated data from Spain, a country with notably high depression rates^26^. This study introduces a compartmental model with seven compartments, capturing various stages of depression progression and dividing individuals into primary and secondary categories. Notably, this model is innovative as it integrates both pharmacological and non-pharmacological treatment strategies, incorporates real-time data to adjust parameters dynamically, and employs a multifactorial approach that considers the impact of the Saboteurs population on depression transmission. By highlighting the role of the Saboteurs population in increasing depression among individuals, this comprehensive framework enables the examination of the effects of different interventions while focusing on reducing the spread of depression and improving access to treatment and counseling centers^27^. The structure of the paper is as follows: second section outlines the formulation of the model, the existence of equilibrium points, stability analysis, and sensitivity analysis with respect to $R_0$. Third section covers the mathematical analysis of the depression model, while next section discusses optimal control strategies aimed at enhancing treatment and counseling. Finally, we conclude with a summary of our findings and their implications.

## Mathematical model

This compartmental model of depression categorizes individuals into several compartments, each playing a distinct role in understanding the dynamics of the disorder. The compartments include susceptible (*S*) individuals who have not yet developed significant depressive symptoms but may be at risk; (*B*) detractors or saboteurs, defined as individuals who deliberately obstruct or undermine others’ progress and success; individuals in the primary stage of depression compartment (*P*), typically characterized by early onset symptoms such as sadness and lack of motivation^28,29^; individuals in the secondary stage of depression compartment (*D*), often experiencing a more severe and chronic course due to underlying conditions^28,29^; individuals undergoing non-pharmacological treatments (*M*), such as cognitive behavioral therapy and exercise therapy, which are crucial for managing mild-to-moderate depression^30^; individuals undergoing pharmacological treatments (*C*); and individuals in the recovery compartment (*R*) who have successfully managed their depression. The distinction between primary and secondary depression is central to this model. Primary depression refers to the early onset of depressive symptoms, generally less severe, with fewer chronic symptoms and less frequent suicidal behavior^28,29^, whereas secondary depression follows a more severe and prolonged course, often arising from underlying physical or psychological conditions and associated with chronic dysphoria, suicidal thoughts, and poorer treatment outcomes after somatic therapies^28,29^. Although this compartmentalization offers a useful framework for study, it might not fully reflect the subtleties of mental health conditions. Hence, it is important to take into account how the compartments interact. All things considered, the model highlights how crucial these classifications are for comprehending the dynamics of depression and how non-pharmacological therapies might enhance overall outcomes. The following assumptions are made to develop the model:

- $\Lambda$ be the constant recruitment rate at which people enter to susceptible compartment.
- $\beta _1$ be the rate at which susceptible individuals contact with Detractors (or) Saboteurs become Detractors (or) Saboteurs.
- The susceptible individuals become primary depressed individuals with contact with secondary depressed individuals at the rate $\beta _2$.
- The individuals in *P* compartment who are in contact with saboteurs move to secondary depressed individuals at the rate $\lambda _1$.
- The individuals in *P* compartment move to *D* compartment at the rate $\lambda _2$.
- At the rate $\psi _1$, individuals in the primary depressed state transition to the non-pharmacological treatment compartment. These treatments encompass regular physical activity, a balanced diet rich in fruits and vegetables, high-quality sleep, and the practice of yoga and meditation.
- The individuals in the secondary depressed compartment transition to pharmacological treatment at the rate $\psi _2$. These treatments include regulated counseling, therapy, and the administration of antidepressant medications.
- The individuals in *M* and *C* compartments move to recovery compartment at the rate $\gamma _1$ and $\gamma _2$ respectively.
- $\sigma$ be rate at which individuals in the secondary depressed compartment die, including deaths related to severe depression, such as suicide, heart attacks, strokes, and other associated complications^31^.
- $\omega$ represents the relapse rate of individuals who have recovered from depression but later move to secondary depression compartment^32,33^.
- $\mu$ denote the natural death rate for individuals in all compartments.

**Fig. 1:**
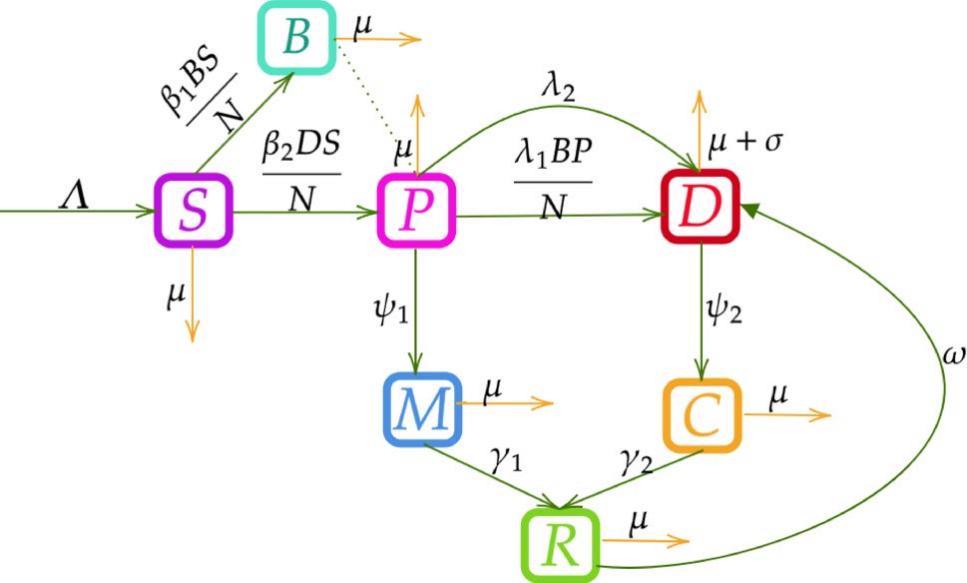
Flow diagram for depression model.

Our suggested compartmental model is represented by the system of differential equations that follows, the above assumptions as describe in Fig. 1. Let *N* be the total population, as provided by

1 $$\begin{aligned} N= & S+B+P+D+M+C+R,\nonumber \\ \frac{dS}{dt}= & \Lambda -\frac{\beta _1 B S}{N} -\frac{\beta _2 D S}{N} -\mu S,\nonumber \\ \frac{dB}{dt}= & \frac{\beta _1 B S}{N} -\mu B,\nonumber \\ \frac{dP}{dt}= & \frac{\beta _2 D S}{N} - \frac{\lambda _1 B P}{N} -(\lambda _2+\mu +\psi _1) P,\nonumber \\ \frac{dD}{dt}= & \frac{\lambda _1 B P}{N} + \lambda _2 P+\omega R-(\psi _2+\mu +\sigma ) D,\nonumber \\ \frac{dM}{dt}= & \psi _1 P -(\gamma _1+\mu ) M,\nonumber \\ \frac{dC}{dt}= & \psi _2 D - (\mu +\gamma _2) C,\nonumber \\ \frac{dR}{dt}= & \gamma _1 M + \gamma _2 C -(\mu +\omega ) R. \end{aligned}$$

## Mathematical analysis of the model

### Positivity and bounded nature of the model solution

From Eq. (1), we obtain the following

$$\begin{array}{llll} \frac{dS}{dt}\Bigr |_ {S=0} = \Lambda >0; & \frac{dB}{dt}\Bigr |_ {B=0} = 0; & \frac{dP}{dt}\Bigr |_ {P=0} = \frac{\beta _2DS}{(S+B+D+M+C+R)} \ge 0 ;& \frac{dM}{dt}\Bigr |_ {M=0}=\psi _1 P \ge 0;\\ \end{array}$$

$$\begin{array}{llll} \frac{dD}{dt}\Bigr |_ {D=0} = \frac{\lambda _1BP}{(S+B+P+M+C+R)}+\lambda _2P +\omega R \ge 0 ;&\frac{dC}{dt}\Bigr |_ {C=0} = \psi _2 D \ge 0;&\frac{dR}{dt}\Bigr |_ {R=0} = \gamma _1 M+ \gamma _2 C \ge 0. \end{array}$$

On the boundary planes, each of the previously mentioned rates is non-negative. As a result, we will eventually stay inside the non-negative bounded cone ${R}_+^7$ if we start there. This is because on all the boundary planes, the vector field direction is inward. It follows that the solution for Eq. (1) will definitely be non-negative. Additionally, we deduce that the entire population of N fulfills the following based on the compartmental model (Eq. 1).

$$\frac{dN}{dt} = \Lambda - \mu N - \sigma D.$$

This gives,

$$\lim \sup \limits _{t\rightarrow \infty } N \le \frac{\Lambda }{\mu }.$$

Therefore, every solutions *S*(*t*), *B*(*t*), *P*(*t*), *D*(*t*), *M*(*t*), *C*(*t*), *R*(*t*) is bounded by $\frac{\Lambda }{\mu }$. This gives us the biologically feasible region of the system (1) by the below positively invariant set:

$$\Omega _1 = \{(S, B, P, D, M, C, R) \in R_+^7 : 0\le S, B, P, D, M, C, R \le \frac{\Lambda }{\mu }\}.$$

### Existence of equilibrium points

In the depression model, there exist four equilibrium points, all of which are non-negative. These equilibrium points represent different situations in how the population behaves and how depression unfolds. To find them, we set the right hand side expressions of the system to zero and solve for the unknowns. This approach helps us pinpoint stable states where the system doesn’t change over time, offering valuable understanding of how depression dynamics play out in the population.

1. At equilibrium point $E_0=(S^*,0,0,0,0,0,0)$, the population comprises only susceptible individuals (*S*), denoted by $S^* = \frac{\Lambda }{\mu }$, with all other variables set to zero,(i.e) $E_0=(\frac{\Lambda }{\mu }, 0, 0, 0, 0, 0, 0)$.
2. The Depression-free equilibrium $E_1 = (S^*, B^*, 0,0,0,0,0)$, where $S^*=\frac{\Lambda }{\beta _1}$ and $B^*=(\frac{\beta _1}{\mu } - 1)\frac{\Lambda }{\beta _1}$ .
3. Saboteurs-free equilibrium $E_2=(S^*, 0, P^*, D^*, M^*, C^*, R^*)$, where
  $$S^* = \frac{(\Lambda a_4 - a_5 a_4)}{\mu } P^*,~~P^* = \frac{\Lambda (1-a_4)}{(\sigma a_4-a_5a_4+a_6 \mu )},~~D^*=a_4 P^*,~~M^*=a_1 P^*$$
  $$C^*=a_2 D^*,~~R=\frac{(\gamma _1 a_1 P^* + \gamma _2 a_2 D^*)}{(\mu +\omega )},~~N=S^*+a_6 P^*$$
  where
  $$a_1=\frac{\psi _1}{(\gamma _1+\mu )},~~a_2=\frac{\psi _2}{(\mu +\gamma _2)},~~a_3=(\psi _2+\mu +\sigma ),~~a_4=\frac{\lambda _2(\mu +\omega )+a_1}{(a_3(\mu +\omega )-a_2)},$$
  $$a_5=(\lambda _2+\mu +psi_1),~~a_6=\frac{a_3a_4}{\lambda _2}+a_4+a_1+a_2a_4+\frac{(\lambda _2-\omega )(\gamma _1a_1+a_2a_4\gamma _2)}{\lambda _2(\mu +\omega )}$$
4. The endemic equilibrium $$E_3 = (S^{**}, B^{**}, P^{**}, D^{**}, M^{**}, C^{**}, R^{**})$$, where
  $$S^{**}=\frac{N\mu }{\beta };~~B^{**}=\frac{A_1 N}{A_2 - A_3 N};~~P^{**} = \frac{b_3 D^{**}N}{(\lambda _1 B^{**} +a_4 N)};~~D^{**}=\frac{(C_1-C_2 N+C_3 N^2)}{(C_4-C_5N)}$$
  $$M^{**}=a_1P^{**};~~C^{**} = a_2 D^{**};~~R^{**}=b_1 P^{**}+b_2D^{**};$$
  and *N* is the positve root of the following equation
  $$W_1 N^5+W_2 N^4+W_3 N^3+W_4 N^2+W_5 N+W_6 = 0.$$
  where
  $$\begin{aligned} W_{1} & = (1 + a_{1} + b_{1} )b_{3} \beta _{1} A_{3}^{2} C_{3} , \\ W_{2} & = (2 + a_{2} + b_{2} + \mu )A_{3}^{2} C_{5} a_{4} - (1 + a_{1} + b_{1} )b_{3} \beta _{1} (2A_{2} A_{3} C_{3} + A_{3}^{2} C_{2} ), \\ W_{3} & = a_{4} A_{3} [\mu (A_{3} C_{4} + A_{2} C_{5} - (a_{3} + C_{5} )] + (\mu - 1)(\lambda _{1} A_{1} + a_{4} A_{2} )A_{3} C_{5} + (1 + a_{1} + b_{1} )b_{3} \beta _{1} \\ & \quad C_{1} A_{3}^{2} + A_{2}^{2} C_{3} + 2A_{2} A_{3} C_{2} ) - (1 + a_{2} + b_{2} )[C_{3} A_{3} (\lambda _{1} A_{1} + a_{4} A_{2} ) + A_{2} A_{3} a_{4} + C_{2} a_{4} A_{3}^{2} ] \\ W_{4} & = (\lambda _{1} A - 1 + a_{4} )(A_{3} + C_{5} - \mu (A_{3} C_{4} + A_{2} C_{5} ) + 3(1 + a_{2} + b_{2} )C_{3} A_{2} ) + a_{4} A_{3} [A_{2} C_{4} - \mu A_{2} C_{4} + A_{1} \beta _{1} C_{5} \\ & \quad + A_{2} (1 + a_{2} + b_{2} ) + C_{1} A_{3} ] - b_{3} \beta _{1} (1 + a_{1} + b_{1} )(2A_{2} A_{3} C_{1} + C_{2} A_{2}^{2} ), \\ W_{5} & = (\lambda _{1} A_{1} + a_{4} A_{2} )[\mu A_{2} C_{4} - A_{2} C_{4} - A_{1} \beta _{1} C_{5} - (1 + a_{2} + b_{2} )(C_{2} A_{2} + C_{1} A_{3} )] - A_{1} \beta _{1} a_{4} A_{3} C_{4} \\ & \quad + (1 + a_{1} + b_{1} )b_{3} \beta _{1} A_{2}^{2} C_{1} - (1 + a_{2} + b_{2} )C_{1} A_{2} A_{3} a_{4} \\ W_{6} & = A_{1} \beta _{1} C_{4} (\lambda _{1} A_{1} + a_{5} A_{2} ) + (1 + a_{2} + b_{2} )A_{2} C_{1} (\lambda _{1} A_{1} + a_{5} A_{2} ). \\ \end{aligned}$$
  $$a_1=\frac{\psi _1}{(\gamma _1+\mu )},~~a_2=\frac{\psi _2}{(\mu +\gamma _2},a_3=(\psi _2+\mu +\sigma ),~~a_4=(\lambda _2+\mu +\psi _1),~~b_1=\frac{\gamma _1 a_1}{(\mu +\omega )},~~b_2=\frac{\gamma _2 a_2}{(\mu +\omega )},~~b_3=\frac{\mu \beta _2}{\beta _1},$$
  $$A_1=a_4a_3\beta _1(a_3-b_2)-(\lambda _2+b_1)\mu \beta _2,~~A_2=\lambda _1a_3(a_3-b_2),~~A_3=\frac{\mu \beta _2\lambda _1}{\beta _1},~~C_1=(\Lambda A_2\beta _1-\mu A_1 \beta _1),$$
  $$C_2=\beta _1A_3\Lambda +\mu ^2A_2,~~C_3=\mu ^2A_3,~~C_4=\beta _1 A_2,~~C_5=\beta _1 A_3.$$

Since determining the exact nature of the roots is challenging, the possible number of positive and negative real roots is summarized in Table 1, based on Descartes’ Rule of Signs.

**Table 1:** Existence of possible roots for $W_1 N^5+W_2 N^4+W_3 N^3+W_4 N^2+W_5 N+W_6 = 0$.

Coefficient signs	Number of positive roots	Number of negative roots
All coefficients are positive	0	5, 3, or 1
Alternating signs: $W_1, W_3, W_5$ positive; $W_2, W_4, W_6$ negative	5, 3, or 1	0
Mixed signs (e.g., $W_1, W_4$ positive; $W_2, W_3, W_5, W_6$ negative)	atmost 3	2 or 0

We can find the endemic equilibrium point $E_3$ provided *N* is positive that leads to positive values for all other variables as expressed before.

### Basic reproduction number

In the depression model, the basic reproduction number ($R_0$) serves as a crucial epidemiological metric, quantifying the average number of new depression cases arising from a single case within a susceptible population. $R_0$ offers crucial insights into the dynamics of depression propagation within communities, particularly considering the influence of Saboteurs. These individuals can negatively impact the mental health of others, thereby contributing to the spread of depression. To determine the basic reproduction number $R_0$ using the next-generation matrix method, we identify the terms representing transmission (${\mathscr {F}}$) and infectiousness (${\mathscr {V}}$) within the context of our depression model. In this framework, we focus on the compartments of individuals as primary depression ($P$), secondary depression ($D$), those undergoing non-pharmacological treatments ($M$), and those receiving pharmacological treatments ($C$). The interactions among these compartments, influenced by the presence of saboteurs, are critical for understanding the overall spread of depression in the population.

$${\mathscr {F}} = \left( \begin{array}{c} \frac{(\beta _2 D S )}{N}\\ 0\\ 0\\ 0 \end{array}\right) ~~\text {and}~~{\mathscr {V}} = \left( \begin{array}{c} (\lambda _2+\mu +\psi _1)P+\frac{\lambda _1 B P}{N} \\ (\psi _2+\mu +\sigma )D-\lambda _2 P -\omega R-\frac{\lambda _1 B P}{N}\\ (\gamma _1+\mu )M-\psi _1 P\\ (\mu +\gamma _2)C-\psi _2 D \end{array}\right)$$

$$F~=~\text {Jacobian of} ~{\mathscr {F}} ~=~\left( \begin{array}{cccc} 0 & \beta _2 & 0 & 0\\ 0 & 0 & 0 & 0\\ 0 & 0 & 0 & 0\\ 0 & 0 & 0 & 0 \end{array}\right)$$

$$V~=~ \text {Jacobian of}~{\mathscr {V}}~=~\left( \begin{array}{cccc} \lambda _2 +\mu +\psi _1 +\frac{\lambda _1 \,{\left( \beta _1 -\mu \right) }}{\beta _1 } & 0 & 0 & 0\\ -\lambda _2 -\frac{\lambda _1 \,{\left( \beta _1 -\mu \right) }}{\beta _1 } & \mu +\psi _2 +\sigma & 0 & 0\\ -\psi _1 & 0 & \gamma _1 +\mu & 0\\ 0 & -\psi _2 & 0 & \gamma _2 +\mu \end{array}\right)$$

and it follows that

$$FV^{-1} ~=~ \left( \begin{array}{cccc} \frac{\beta _2 \,{\left( \beta _1 \,\lambda _1 +\beta _1 \,\lambda _2 -\lambda _1 \,\mu \right) }}{{\left( \mu +\psi _2 +\sigma \right) }\,{\left( \beta _1 \,\lambda _1 +\beta _1 \,\lambda _2 +\beta _1 \,\mu +\beta _1 \,\psi _1 -\lambda _1 \,\mu \right) }} & \frac{\beta _2 }{\mu +\psi _2 +\sigma } & 0 & 0\\ 0 & 0 & 0 & 0\\ 0 & 0 & 0 & 0\\ 0 & 0 & 0 & 0 \end{array}\right)$$

The eigenvalues of $FV^{-1}$ which is the largest is the basic reproduction number $R_0$ and here we obtain six of the eigenvalues to be 0 and the other non-zero eigenvalue is the largest which is given below:

$$R_0 = \frac{\beta _2 \,{\left( \beta _1 \,\lambda _1 +\beta _1 \,\lambda _2 -\lambda _1 \,\mu \right) }}{{\left( \mu +\psi _2 +\sigma \right) }\,{\left( \beta _1 \,\lambda _1 +\beta _1 \,\lambda _2 +\beta _1 \,\mu +\beta _1 \,\psi _1 -\lambda _1 \,\mu \right) }}.$$

### Stability analysis

#### Theorem 1

The equilibrium point $E_0~=~(\frac{\Lambda }{\mu },0,0,0,0,0,0)$ is locally asymptotically stable under some restriction on parameters.

#### Proof

The proof of the theorem is available in the supplementary document. $\square$

#### Theorem 2

The depression-free equilibrium point $E_1 = (S^*, B^*, 0,0,0,0,0)$, where $S^*=\frac{\Lambda }{\beta _1}$ and $B^*=(\frac{\beta _1}{\mu } - 1)\frac{\Lambda }{\beta _1}$ is locally asymptotically stable under some restriction on parameters.

#### Proof

The proof of the theorem is available in the supplementary document. $\square$

#### Theorem 3

The saboteurs-free equilibrium point $E_2 = (S^*, 0, P^*, D^*, M^*, C^*, R^*)$, is locally asymptotically stable under some restriction on parameters.

#### Proof

The proof of the theorem is available in the supplementary document. $\square$

#### Theorem 4

The Endemic equilibrium point $$E_3 = (S^{**}, B^{**}, P^{**}, D^{**}, M^{**}, C^{**}, R^{**})$$, is locally asymptotically stable under some restriction on parameters.

#### Proof

The proof of the theorem is available in the supplementary document. $\square$

## Numerical simulation

The model solution, data fitting, parameter estimations, and depression prevalence are among the results from executing different simulations that are shown in this section. These results have been discussed in detail in this section. For depression or depressive disorder data from Spain, the model calibration is complete.

### Data fitting

To capture the intricacies of depression, our approach relies on data fitting to uncover trends and patterns within complex datasets. By integrating real-world data into our mathematical models such as demographic profiles, clinical assessments, and environmental factors we aim to enrich our understanding of depression dynamics. We leverage statistical methods like Maximum Likelihood Estimation (MLE), as detailed in ^37^, and implement them in Python to tailor our models to accurately reflect the multifaceted interplay between these variables.

Specifically, we estimate the transmission rate of depression ($\beta _2$), the influence of saboteurs ($\beta _1$), and the recovery rate ($\gamma _2$), while other parameters are drawn from literature reviews or assumed based on realistic scenarios. The constant recruitment rate ($\Lambda$) is calculated from the birth rate of individuals in Spain, and the natural death rate ($\mu$) is determined by the reciprocal of the average lifespan of individuals in Spain (80 years). The results indicate variability in the estimated parameters, reflecting their sensitivity to the underlying data. The 95% confidence intervals provide valuable insights into the precision of these estimates.

**Fig. 2:**
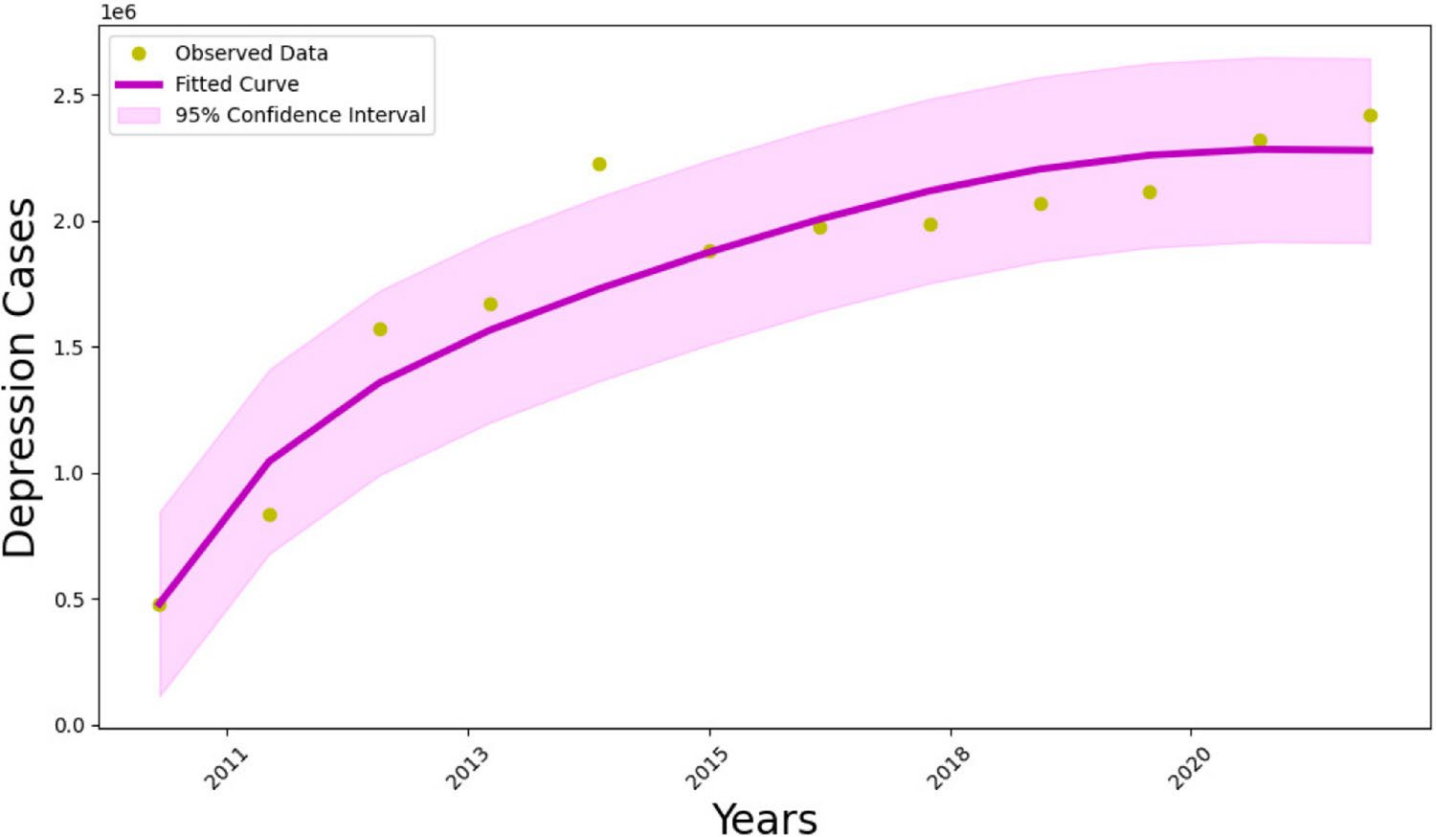
Graphs showing the model’s fit with real depression disorder cases in Spain. The yellow dots show the actual data, and the magenta line shows the model’s solution.

Overall, the model provides a satisfactory fit to the data, as depicted in Fig. 2, with yellow dots representing actual data and the magenta line representing the model’s solution. For the yearly period from 2011 to 2022, we scrutinized depression data from Spain^38^, fitting our proposed model to achieve a good match. The MLE method provided realistic values for the parameters, confirming the model’s viability, as illustrated in Fig. 2, Tables 2 and 3.

**Table 2:** Parameters values.

Parameter	Value	References
$\Lambda$	7.816/10,000	^34^
$\lambda _1$	0.2145	Assumed
$\lambda _2$	0.7	^35^
$\psi _1$	0.0045	Assumed
$\gamma _1$	0.6	^36^
$\sigma$	0.01	^15^
$\omega$	0.005	Assumed
$\psi _2$	0.02	Assumed
$\mu$	0.0125	Demographic

**Table 3:** Parameters values and their confidence intervals.

Parameter	Value	95% confidence interval	Value of $R_0$
$\beta _1$	0.5665	0.4685–0.6645	
$\beta _2$	0.1567	0.0587–0.2547	3.6219
$\gamma _2$	0.9219	0.8827–0.9611	

### Sensitivity analysis

In epidemiological research, sensitivity analysis is an essential tool because it shows how changes in important factors, including those that affects the reproduction number $R_0$, affect the study’s results. This method guarantees a comprehensive assessment of the study’s validity and strengthens the findings’ credibility by emphasizing how sensitive the results are to these important elements. In our depression model, the key parameters $\beta _1, \beta _2, \lambda _1, \lambda _2, \psi _1, \psi _2$ significantly affect the basic reproduction number $R_0$. In this study, we employ the forward sensitivity index to conduct specific analyses, which is calculated by examining the relative change in a variable concerning a given parameter. This sensitivity can be expressed in terms of partial derivatives if the variable is a differentiable function of the specified parameter, a method applied in our work^39,40^.

**Fig. 3:**
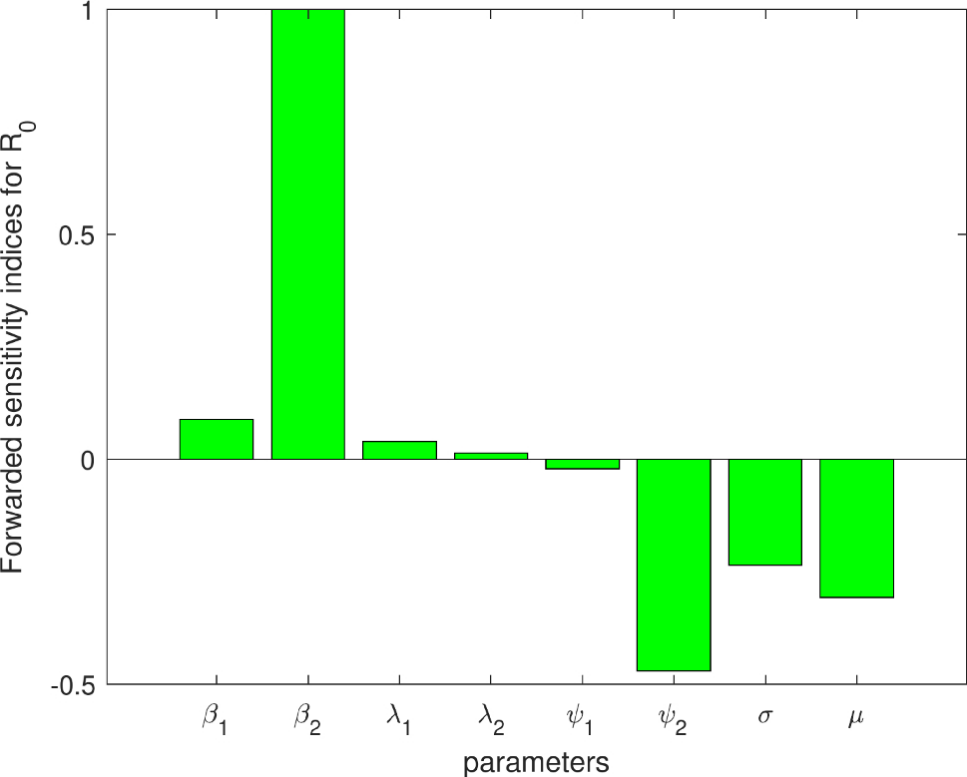
Normalized forward sensitivity index of the basic reproduction number of the depression model. The parameter values are as in Table 2.

From Fig. 3, we found that $\beta _1, \beta _2, \lambda _1, \lambda _2$ have positive indices associated with the basic reproduction number $R_0$, indicating that these parameters play a vital role in increasing the depression rate. Conversely, the parameters $\psi _1, \psi _2, \sigma$, and $\mu$ show negative correlations with $R_0$, as depicted in Fig. 3. This suggests that enhancing treatment rates and implementing early detection measures can effectively mitigate the spread of the depression.

**Fig. 4:**
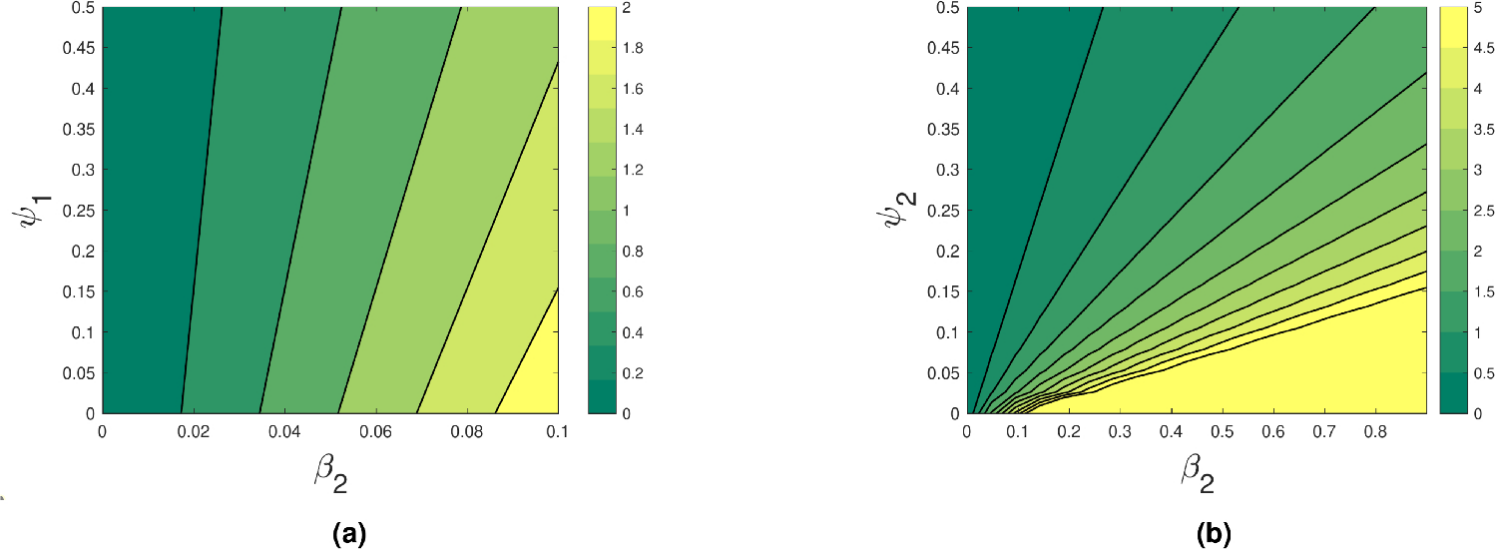
Contour plots showing the effect of (**a**) $\beta _2$ and $\psi _1$ and (**b**) $\beta _2$ and $\psi _2$ on the basic reproduction number.

**Fig. 5:**
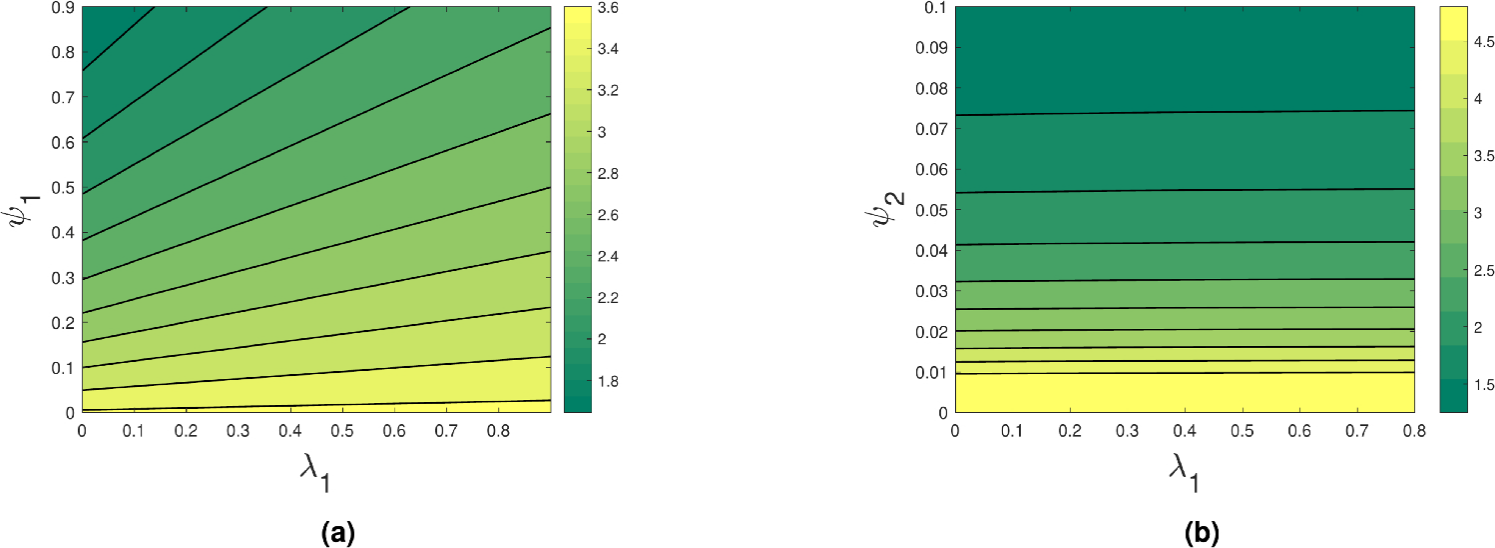
Contour plots showing the effect of (**a**) $\lambda _1$ and $\psi _1$ and (**b**) $\lambda _1$ and $\psi _2$ on the basic reproduction number.

**Fig. 6:**
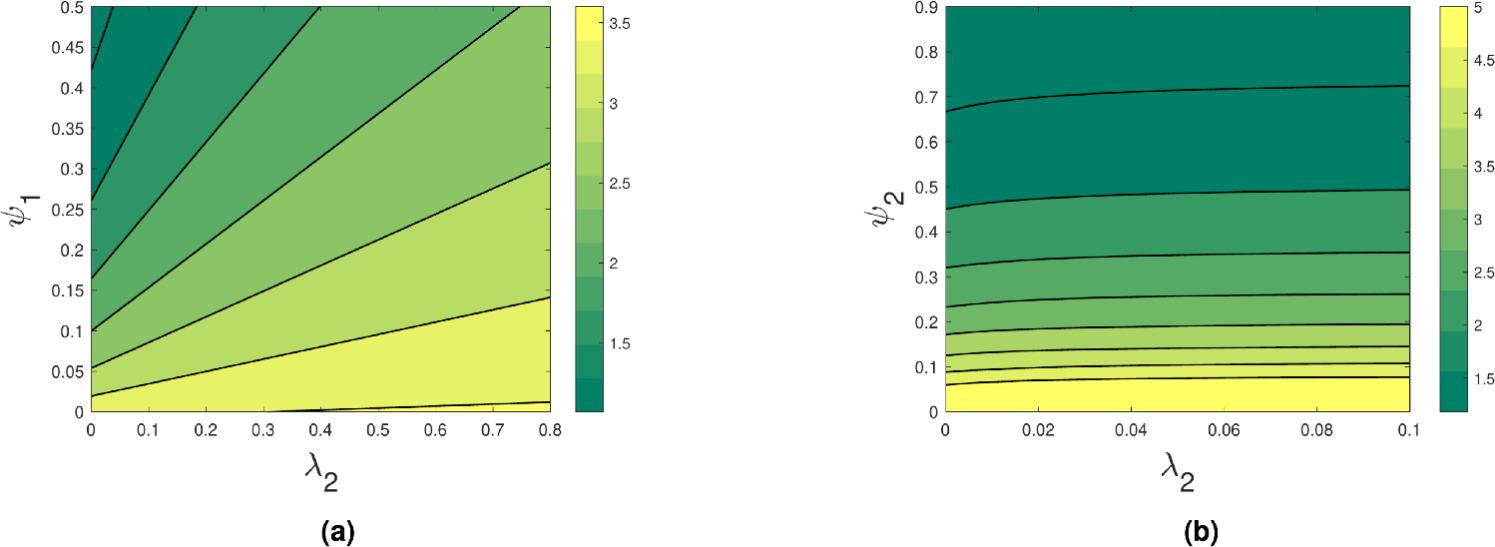
Contour plots showing the effect of (**a**) $\lambda _2$ and $\psi _1$ and (**b**) $\lambda _2$ and $\psi _2$ on the basic reproduction number.

By using two parameters at once, the contour plot in Fig. 4 shows how the reproduction number value varies. These data points provide a more visual comprehension of the rise and fall of the $R_0$ value with respect to the major parameters. Figure 4 shows that whereas $R_0$ value rises with increasing $\beta _2$ values, they fall with increasing $\psi _1$ and $\psi _2$. Figures 5 and 6 show that as $\lambda _1,~\lambda _2$ increase, $R_0$ value increases and decreases when $\psi _1,~\psi _2$ increase. This shows that increasing treatment and early detection helps to control the spread of depression.

### Impact of various factors on depression prevalence

**Fig. 7:**
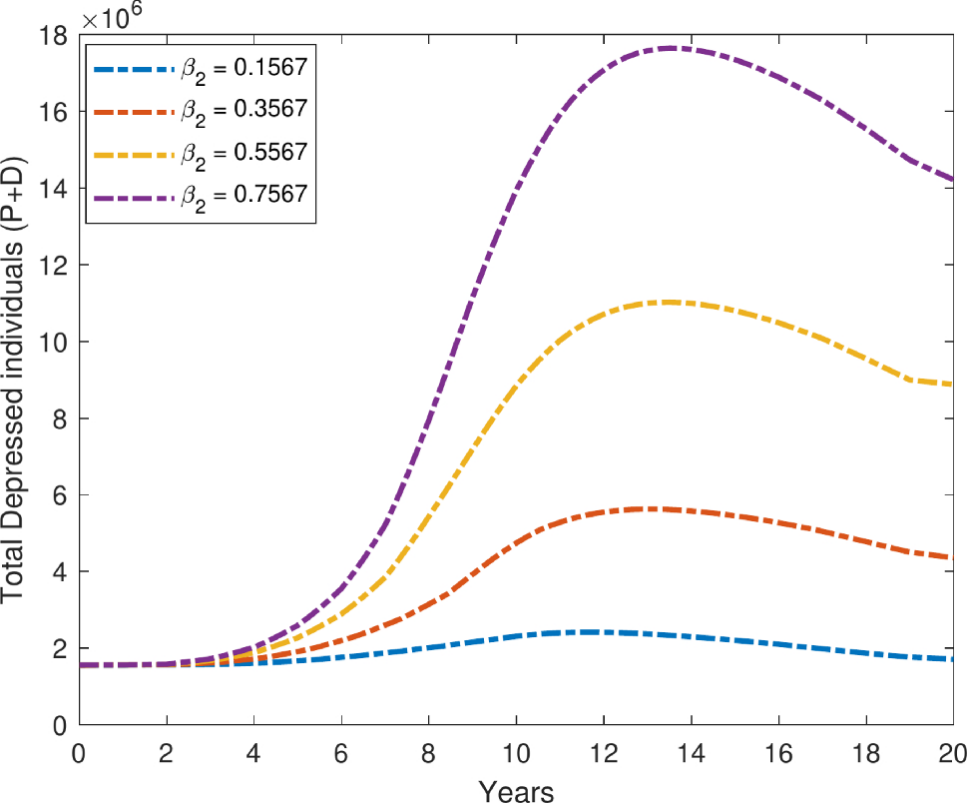
Variation in total depressed individuals with respect to $\beta _2$.

**Fig. 8:**
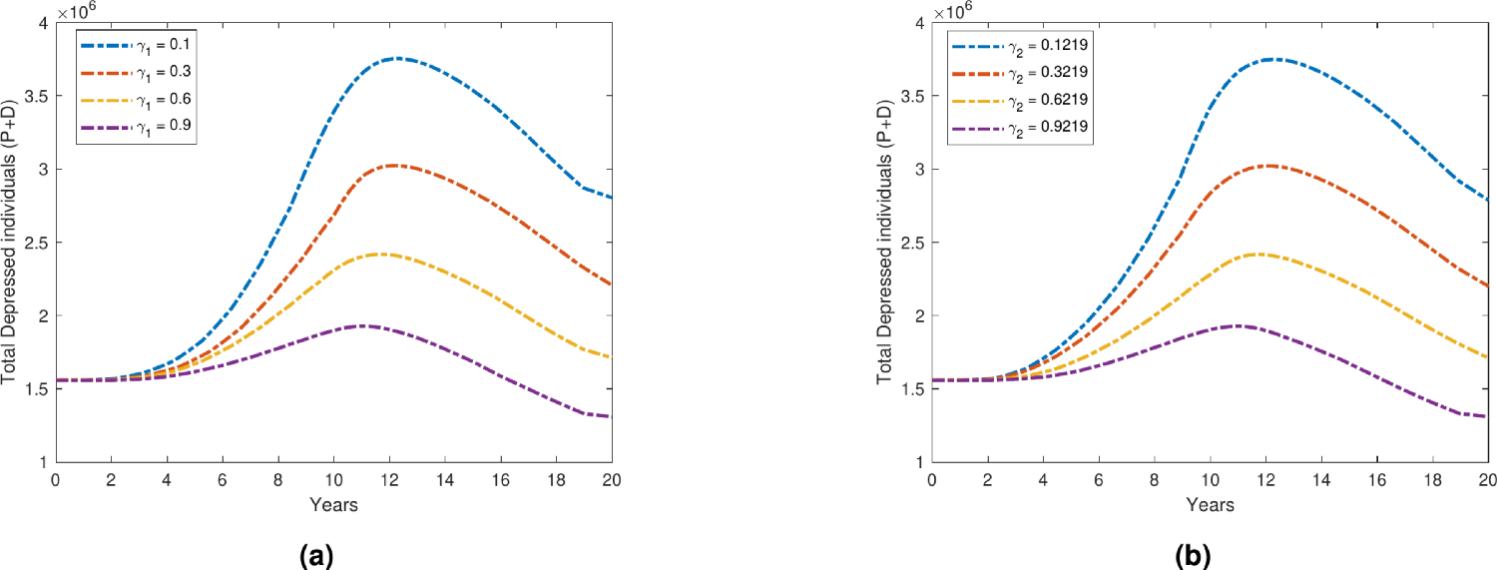
Variation in Total depressed individuals with respect to (**a**)$\gamma _1$; (**b**) $\gamma _2$.

**Fig. 9:**
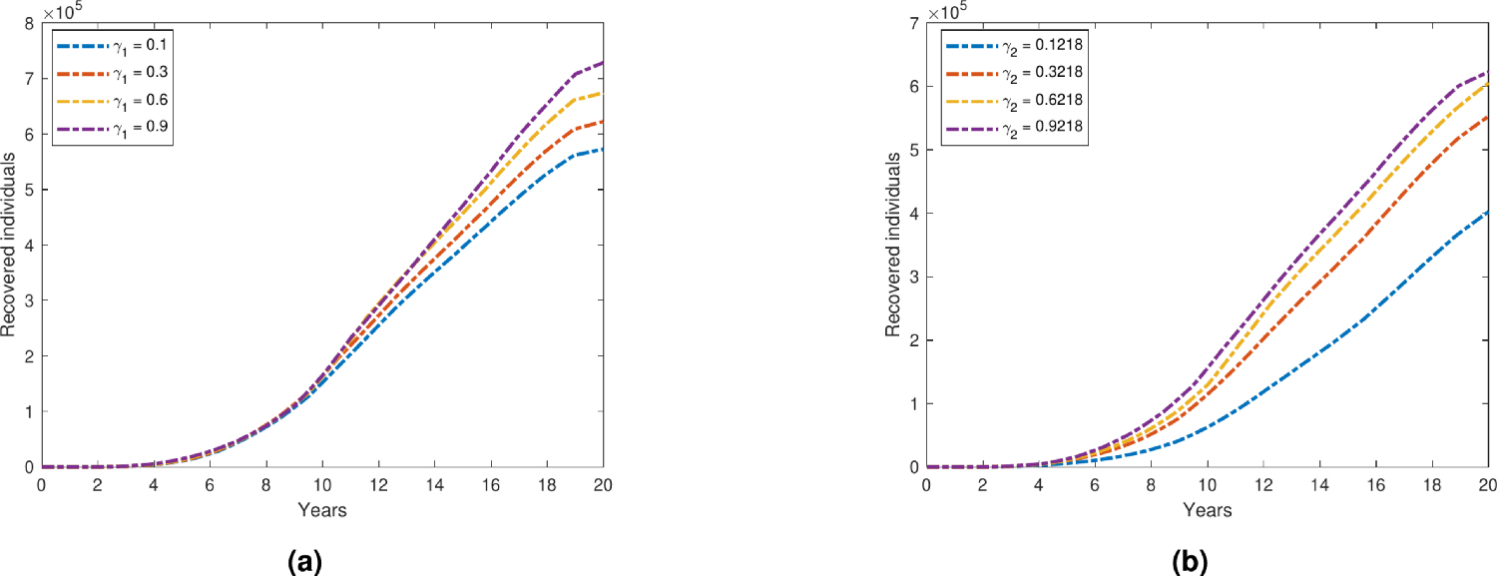
Variation in Recovered individuals with respect to (**a**)$\gamma _1$; (**b**) $\gamma _2$.

We examine the time evolution plot of our proposed model (1) over a 20-year period for the depression compartment, with a variable transmission rate $\beta _2$. Figure 7 illustrates the effect of changes in the transmission rate on the total number of individuals experiencing depression during this prolonged period. As the value of $\beta _2$ increases, there is a significant rise in the number of individuals in total depressed compartments. This suggests that higher transmission rates lead to a greater spread of depression within the population. Notably, the depression curve exhibits a steeper increase as $\beta _2$ increases, indicating that small changes in the transmission rate have a substantial impact on the overall depression dynamics. On the other hand, Fig. 8 illustrates how increasing the recovery rates $\gamma _1$ and $\gamma _2$ affects the overall number of people who suffer from depression. As anticipated, the number of people in the primary and secondary depressed compartments clearly decreases as recovery rates rise. This is in line with the theory that faster recovery from depression is associated with higher $\gamma$ values, which in turn lowers the total number of persons who experience depression over time. The decline becomes more noticeable as the time progresses, suggesting that improved recovery rates have a cumulative impact on the population’s long-term dynamics of depression. Figure 9 further supports this finding by illustrating the inverse relationship between the recovery rate $\gamma$ and the number of depressed individuals. As $\gamma$ increases, the duration of the depression period shortens, leading to a faster transition of individuals from the depressed state to recovery. Consequently, the total number of people with depression decreases over time. The figure also highlights that as recovery rates increase, the number of recovered individuals rises significantly. This correlation reinforces the notion that faster recovery rates promote a quicker transition to a recovered state, ultimately decreasing the overall burden of depression in the population.

### Impact of treatment on depression prevalence

**Fig. 10:**
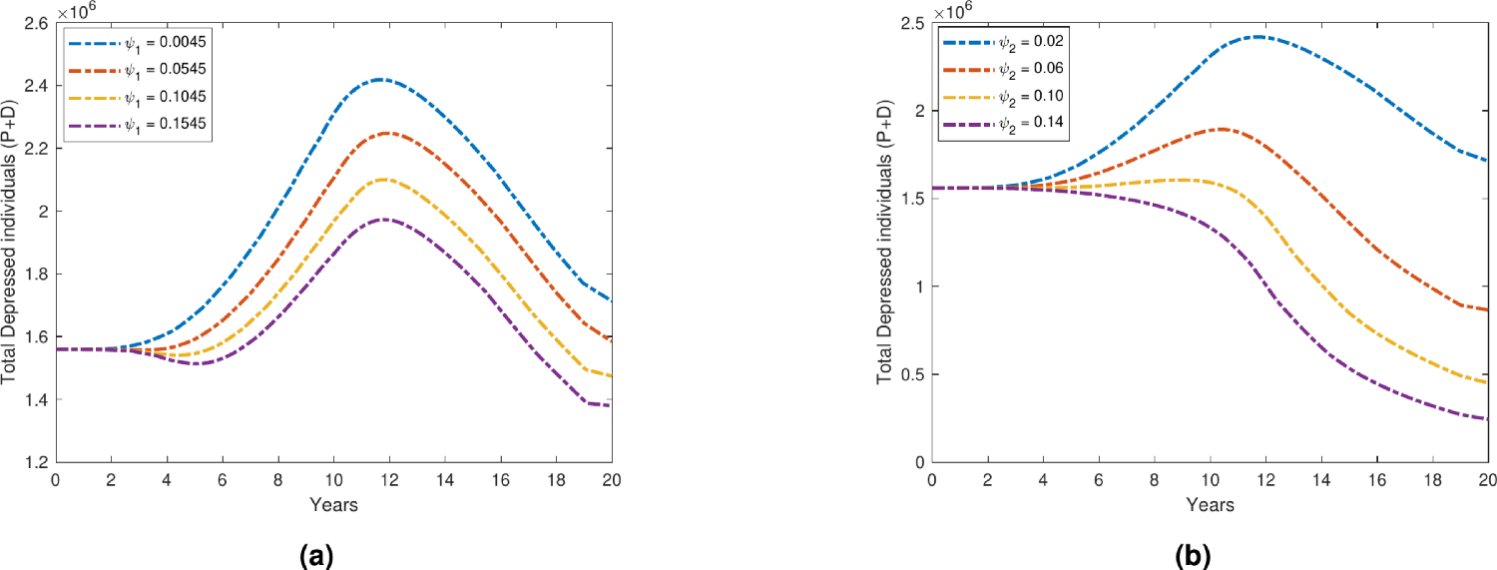
Variation in total depressed individuals with respect to (**a**)$\psi _1$; (**b**) $\psi _2$.

Figure 10 shows that the overall number of depressed individuals declines when we increase the treatment and early detection rates ($\psi _1$ and $\psi _2$), which stand for self-medication and seeking treatment, respectively. This implies that the prevalence of depression is lowered as a result of both self-medication and seeking consulting services.

**Fig. 11:**
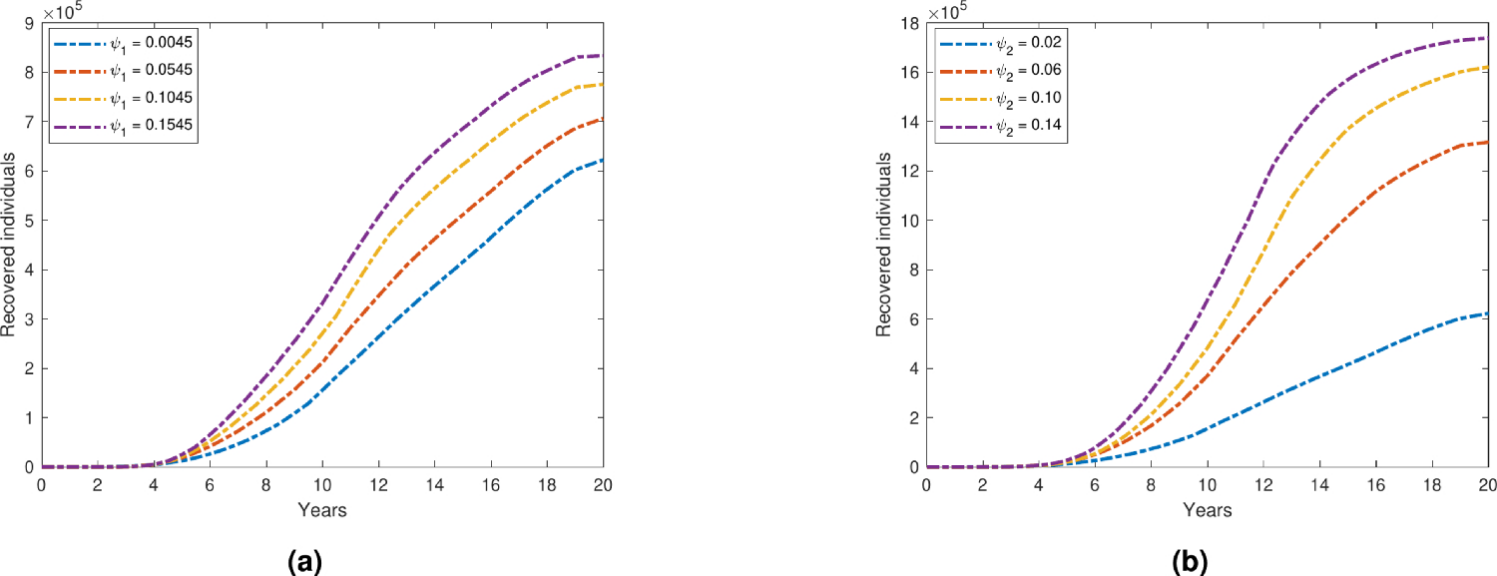
Variation in recovered individuals with respect to (**a**) $\psi _1$; (**b**) $\psi _2$.

Additionally, as the treatment and early detection rates ($\psi _1$ and $\psi _2$) rise, Fig. 11 shows that the recovery compartment rises similarly. This suggests that more people who self-medicate and see a doctor have better outcomes while recovering from depression.

The necessity of a comprehensive approach to mental health care is emphasized by these findings, which emphasize the role of professional medical intervention as well as self-care practices in the management and treatment of depression.

### Impact of interaction with saboteurs on depression prevalence

**Fig. 12:**
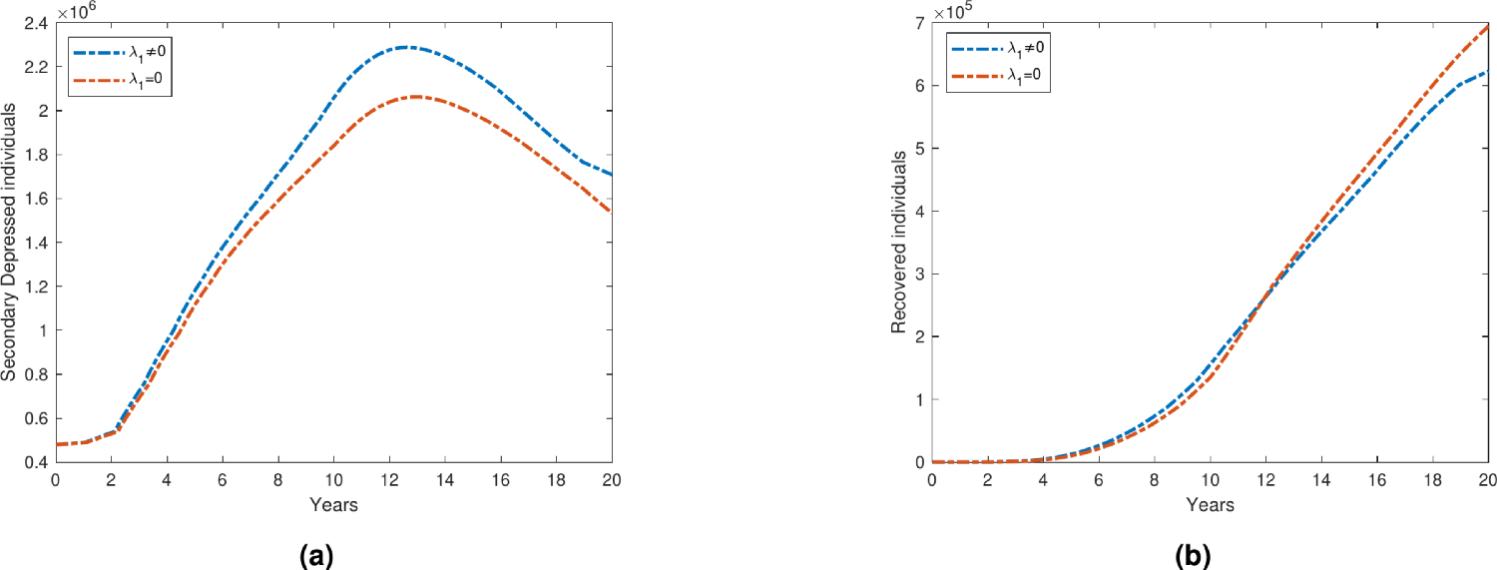
With and without interaction of Saboteurs.

A population of saboteurs is included in our proposed compartment model, and $\lambda _1$ represents the rate at which primary depressed individuals who interact with saboteurs transition to the secondary depressed compartment. Figure 12 compares the dynamics of the depression compartments under two scenarios: one with interaction between the population and saboteurs, and one without such interaction. In the scenario where individuals interact with saboteurs, the number of people transitioning from the primary to the secondary depressed compartment rises significantly over time. This is because the influence of saboteurs accelerates the decline in mental health among those already depressed, pushing them into a more severe state. As shown in Fig. 12, the slope of the curve corresponding to the secondary depressed compartment is steeper when the interaction rate $\lambda _1$ is higher, indicating a faster progression into more severe depression due to the presence of saboteurs.

In contrast, when there is no communication with saboteurs, the overall number of depressed individuals in both compartments decreases at a much faster rate. This suggests that avoiding interaction with saboteurs can have a protective effect on mental health. The absence of saboteurs not only slows the rate of transition into secondary depression but also promotes a higher rate of recovery in both compartments. This is particularly evident as the number of individuals in the recovered group increases more rapidly in the no-saboteur scenario. Figure 12 illustrates that minimizing or eliminating exposure to negative influences such as saboteurs can lead to better mental health outcomes, as evidenced by lower depression rates and higher recovery rates in the no-contact scenario.

## Optimal control

Optimal control is an invaluable mathematical tool that may be employed to control the propagation of depression within a population. The primary objective of this research is to identify the most effective control strategies that can be implemented with minimal cost. In numerous epidemiological problems, optimal control has been demonstrated to be a successful approach. In this section, we expand the mathematical model outlined in Eq. (1) by introducing control parameters to formulate an optimal control problem with three control parameters, denoted as $u_1$, $u_2$ and $u_3$ associated with identifying the primary depressed individuals and providing treatment, identifying the secondary depressed individuals and providing treatment and enhancing treatment, respectively.

The control variable $u_1$ identifies individuals with primary-stage depression and offers non-pharmacological interventions. These treatments encompass regular physical activity like exercise^41^, a balanced diet abundant in fruits and vegetables, high-quality sleep, and the practice of yoga and meditation. Mindfulness-based stress reduction (MBSR) and cognitive behavior therapy (CBT) is a structured approach aimed at alleviating stress^42^. Such interventions are aimed at mitigating depression among individuals in the primary stage, thereby reducing the prevalence of depression in this population.

The control parameter $u_2$ identifies individuals facing secondary-stage depression and applies pharmacological treatments. These treatments encompass regulated counseling, therapy, and the use of antidepressant medications such as SSRIs, TCAs, MAOIs, and various other antidepressants. Nonetheless, it presents adverse effects including weight gain, dry mouth, sweating, anxiety, and a heightened risk of suicide^43^. This approach aims to reduce depression in secondary depressed individuals.

The control parameter $u_3$ signifies enhanced treatment approaches, which encompass delivering quality counseling, dedicating time to patients, and arranging appointments according to the severity of their condition, such as MCID (Minimal Clinically Important Difference) and PHQ-9 (patient depression questionnaire)^44^, as well as consistently monitoring individuals through wearables and mobile devices to assess and track depression severity^45^. This all guarantees compliance with their depression medication schedule. These initiatives aim to support the prompt recovery of those impacted. By emphasizing individualized care and ongoing assessment, healthcare professionals can more effectively customize treatment strategies to address the unique requirements of each patient. This method ultimately results in better results and an enhanced quality of life for individuals dealing with depression.

Adopting a comprehensive approach to depression control necessitates incorporating all three measures, $u_1$, $u_2$, and $u_3$. Collectively, these three control parameters play a vital role in controlling and managing the spread of depression. The following is the optimal control problem extended from model (Eq. 1)

2 $$\begin{aligned} {\frac{dS}{dt}}= & {\Lambda -\frac{\beta _1 B S}{N}-\frac{\beta _2 D S }{N}-\mu S},\nonumber \\ {\frac{dB}{dt}}= & {\frac{\beta _1 B S}{N}-\mu B},\nonumber \\ {\frac{dP}{dt}}= & {\frac{\beta _2 D S }{N}-\frac{\lambda _2 B P }{N}}-(\lambda _2+\mu +\psi _1+u_1(t))P,\nonumber \\ {\frac{dD}{dt}}= & {\frac{\lambda _2 B P }{N}}+\lambda _2 P+\omega R-(\psi _2+u_2(t)+\mu +\sigma )D,\nonumber \\ {\frac{dM}{dt}}= & {(\psi _1+u_1(t))P-(\gamma _1+\mu )M},\nonumber \\ {\frac{dC}{dt}}= & {(\psi _2+u_2(t))D-(\gamma _2+u_3(t)+\mu )C},\nonumber \\ {\frac{dR}{dt}}= & {\gamma _1 M +(\gamma _2+u_3(t))C-(\mu +\omega ) R}. \end{aligned}$$

The objective functional for the fixed time $t_f$ is given by:

$$J= \int _{0}^{t_f} (W_1P + W_2D + W_3 M + W_4 C + \frac{1}{2}W_5u_1^2+\frac{1}{2}W_6u_2^2 + \frac{1}{2}W_7u_3^2)dt,$$

where $W_1, W_2, W_3, W_4, W_5, W_6, W_7$ $\ge$0 are seven weight constants.

The objective is to ascertain the control parameters $u_1^*, u_2^*, u_3^*$, such that

$$J(u_1^*,u_2^*, u_3^*)=min J(u_1,u_2,u_3).$$

Here, $\Omega _1$ is the defined control set.

$\Omega _1$ = { $u_1$, $u_2$, $u_3$ : measurable and $a_i\le$ $u_i$, $<b_i$,   for  $i=1,2,3$ } and $t \in [0, t_f ]$

The Lagrangian of this problem is:

$$L(P, D, M, C, u_1, u_2, u_3) = W_1P + W_2D + W_3M + W_4C + \frac{1}{2}W_5u_1^2+\frac{1}{2}W_6u_2^2 + \frac{1}{2}W_7u_3^2$$.

The Hamiltonian ${\mathscr {H}}$ formulated for our problem is,

$${\mathscr {H}}=L(P, D, M, C, u_1, u_2, u_3)+\Lambda _1 \frac{dS}{dt}+\Lambda _2 \frac{dB}{dt}+\Lambda _3 \frac{dP}{dt}+\Lambda _4 \frac{dD}{dt}+\Lambda _5 \frac{dM}{dt}+\Lambda _6 \frac{dC}{dt}+\Lambda _7 \frac{dR}{dt}.$$

Where, $\Lambda _i$’s represents the adjoint variables for $(i=1,\ldots ,7)$.

The results from^46^ assure that an optimal control problem exists. The adjoint variables are represented in the form of differential equations as follows:

3 $$\begin{aligned} \frac{{d\Lambda _{1} }}{{dt}} & = - \frac{{\partial {\mathcal{H}}}}{{\partial S}} = \left( {\frac{{\beta _{1} B}}{{N^{2} }}(N - S)} \right)(\Lambda _{1} - \Lambda _{2} ) + \left( {\frac{{\beta _{2} D}}{{N^{2} }}(N - S)} \right)(\Lambda _{1} - \Lambda _{3} ) - \frac{{\lambda _{1} BP}}{{N^{2} }}(\Lambda _{3} - \Lambda _{4} ) + \Lambda _{1} \mu , \\ \frac{{d\Lambda _{2} }}{{dt}} & = - \frac{{\partial {\mathcal{H}}}}{{\partial B}} = \left( {\frac{{\beta _{1} S}}{{N^{2} }}(N - B)} \right)(\Lambda _{1} - \Lambda _{2} ) + \left( {\frac{{\lambda _{1} P}}{{N^{2} }}(N - B)} \right)(\Lambda _{3} - \Lambda _{4} ) - \frac{{\beta _{2} DS}}{{N^{2} }}(\Lambda _{1} - \Lambda _{3} ) + \Lambda _{2} \mu , \\ \frac{{d\Lambda _{3} }}{{dt}} & = - \frac{{\partial {\mathcal{H}}}}{{\partial P}} = \frac{{\beta _{1} BS}}{{N^{2} }}(\Lambda _{1} - \Lambda _{2} ) - \frac{{\beta _{2} DS}}{{N^{2} }}(\Lambda _{1} - \Lambda _{3} ) + \lambda _{2} (\Lambda _{3} - \Lambda _{4} ) - \left( {\frac{{\lambda _{1} B}}{{N^{2} }}(N - P)} \right)(\Lambda _{3} - \Lambda _{4} ) \\ & \quad + (\psi _{1} + u_{1} (t))(\Lambda _{3} - \Lambda _{5} ) + \mu \Lambda _{3} - W_{1} , \\ \frac{{d\Lambda _{4} }}{{dt}} & = - \frac{{\partial {\mathcal{H}}}}{{\partial D}} = \frac{{\beta _{1} BS}}{{N^{2} }}(\Lambda _{1} - \Lambda _{2} ) + \left( {\frac{{\beta _{2} S}}{{N^{2} }}(N - D)} \right)(\Lambda _{1} - \Lambda _{3} ) - \frac{{\lambda _{1} BP}}{{N^{2} }}(\Lambda _{3} - \Lambda _{4} ) \\ & \quad + (\psi _{2} + u_{2} (t))(\Lambda _{4} - \Lambda _{5} ) + (\mu + \sigma )\Lambda _{4} - W_{2} , \\ \frac{{d\Lambda _{5} }}{{dt}} & = - \frac{{\partial {\mathcal{H}}}}{{\partial M}} = \frac{{\beta _{1} BS}}{{N^{2} }}(\Lambda _{1} - \Lambda _{2} ) - \frac{{\beta _{2} DS}}{{N^{2} }}(\Lambda _{1} - \Lambda _{3} ) - \frac{{\lambda _{1} BP}}{{N^{2} }}(\Lambda _{3} - \Lambda _{4} ) + \gamma _{1} (\Lambda _{5} - \Lambda _{7} ) + \mu \Lambda _{5} - W_{3} , \\ \frac{{d\Lambda _{6} }}{{dt}} & = - \frac{{\partial {\mathcal{H}}}}{{\partial C}} = - \frac{{\partial H}}{{\partial M}} = \frac{{\beta _{1} BS}}{{N^{2} }}(\Lambda _{1} - \Lambda _{2} ) - \frac{{\beta _{2} DS}}{{N^{2} }}(\Lambda _{1} - \Lambda _{3} ) - \frac{{\lambda _{1} BP}}{{N^{2} }}(\Lambda _{3} - \Lambda _{4} ) \\ & \quad + (\gamma _{2} + u_{3} (t))(\Lambda _{6} - \Lambda _{7} ) + \mu \Lambda _{6} - W_{4} , \\ \frac{{d\Lambda _{7} }}{{dt}} & = - \frac{{\partial {\mathcal{H}}}}{{\partial R}} = \frac{{\beta _{1} BS}}{{N^{2} }}(\Lambda _{1} - \Lambda _{2} ) - \frac{{\beta _{2} DS}}{{N^{2} }}(\Lambda _{1} - \Lambda _{3} ) - \frac{{\lambda _{1} BP}}{{N^{2} }}(\Lambda _{3} - \Lambda _{4} ) + \mu \Lambda _{7} + \omega (\Lambda _{7} - \Lambda _{4} ). \\ \end{aligned}$$

Let $\tilde{S}$, $\tilde{B}$, $\tilde{P}$, $\tilde{D}$, $\tilde{M}$, $\tilde{C}$ and $\tilde{R}$ be a optimal value of *S*, *B*, *P*, *D*, *M*, *C* and *R* respectively. Let $\tilde{\Lambda _1}$, $\tilde{\Lambda _2}$, $\tilde{\Lambda _3}$, $\tilde{\Lambda _4}$, $\tilde{\Lambda _5}$, $\tilde{\Lambda _6}$ and $\tilde{\Lambda _7}$ be solutions of Eq. (2), using^47,48^ we present and demonstrate the following theorem.

### Theorem 5

Optimal controls $u_1^*, u_2^*, u_3^*$ belonging to set $\Omega _1$ exist, such that $J(u_1^*,u_2^*,u_3^*)=\min J(u_1,u_2,u_3)$ subject to the extended system of Eq. (2).

### Proof

We utilize^47^ to establish this theorem. In this scenario, it is evident that the controls are non-negative. The necessary convexity of the objective functional in ($u_1$, $u_2$, $u_3$) is satisfied for the minimization problem. A set of control variables such as $u_1$, $u_2$, and $u_3$ within $\Omega _1$ is both convex and closed by definition. The state variables are bounded, and the integrand of the functional

$$W_1P + W_2D + W_3M + W_4C + \frac{1}{2}W_5u_1^2+\frac{1}{2}W_6u_2^2 + \frac{1}{2}W_7u_3^2$$

is convex on $\Omega _1$.

As optimal controls exist for minimizing the functional subject to systems (2) and (3), we employ Pontryagin’s maximum principle to derive the necessary conditions for finding the optimal solutions in the following manner:

Suppose (*z*, u) is an optimal solution of an optimal control problem; then, this implies the existence of a non-trivial vector function $\Lambda = \Lambda _1, \Lambda _2,\ldots , \Lambda _7$ satisfying the following:

$$\frac{dz}{dt}=\frac{\partial {\mathscr {H}}(t,z,u,\Lambda )}{\partial \Lambda },0=\frac{\partial {\mathscr {H}}(t,z,u,\Lambda )}{\partial u},\frac{d\Lambda }{dt}=\frac{\partial {\mathscr {H}}(t,z,u,\Lambda )}{\partial z}.$$

$$\square$$

### Theorem 6

The optimal controls $u_1^*,u_2^*,u_3^*$ that minimize J over the region $\Omega _1$ are expressed as:

$$u_1^* = min\biggr \{b_1,max(a_1,\tilde{u_1})\biggr \},u_2^* = min\biggr \{b_2,max(a_2,\tilde{u_2})\biggr \},u_3^* = min\biggr \{b_3,max(a_3,\tilde{u_3})\biggr \},$$

where,

$${\tilde{u}}_1 = \frac{(\Lambda _3 -\Lambda _5) P}{W_5},{\tilde{u}}_2 = \frac{(\Lambda _4-\Lambda _6) D}{W_6}, {\tilde{u}}_3 = \frac{(\Lambda _6-\Lambda _7) C}{W_7}.$$

### Proof

We establish this theorem by utilizing^47^ and Theorem 5.

Applying the optimality condition: $\frac{\partial {\mathscr {H}}}{\partial u_1}=0 , \frac{\partial {\mathscr {H}}}{\partial u_2}=0, \frac{\partial {\mathscr {H}}}{\partial u_2}=0$, we get,

$$\frac{\partial {\mathscr {H}}}{\partial u_1}= (\Lambda _5 - \Lambda _3) P + W_5 u_1 = 0$$

$$\implies u_1 = \frac{(\Lambda _3 -\Lambda _5) P}{W_5} = {\tilde{u}}_1$$

$$\frac{\partial {\mathscr {H}}}{\partial u_2}=W_6 u_2+(\Lambda _6-\Lambda _4)D = 0$$

$$\implies u_2 = \frac{(\Lambda _4-\Lambda _6) D}{W_6} = {\tilde{u}}_2$$

$$\frac{\partial {\mathscr {H}}}{\partial u_3}=W_7 u_3+(\Lambda _7-\Lambda _6)C = 0$$

$$\implies u_3 = \frac{(\Lambda _6-\Lambda _7) C}{W_7} = {\tilde{u}}_3$$

The controls $u_i$ have a lower bound of $a_i$ and an upper bound of $b_i$. This suggests that $u_i=a_i$ if ${\tilde{u}}_i<a_i$, also $u_i =b_i$ if ${\tilde{u}}_i > b_i$, for $i=1,2,3$ otherwise $u_1={\tilde{u}}_1$ , $u_2={\tilde{u}}_2$ , $u_3={\tilde{u}}_3$ . Hence, for these controls $u_1^*,u_2^*,u_3^*$, we obtain optimal values for J. $\square$

### Numerical simulation of optimal control problem

The optimal control problem is simulated using MATLAB for 15 years. The weight constants are $W_1=1, W_2=1,W_3=1,W_4=1,W_5=15,W_6=40,W_7=55$, and for simulation we have chosen $a_i=0$ and $b_i=1$ for $i=1,2,3$. The extended system of Eq. (2) is iteratively solved using forward and backward difference approximations^48^. In the following subsections, we focus on the impact of different control strategies.

#### Strategy A: $u_1$ only

**Fig. 13:**
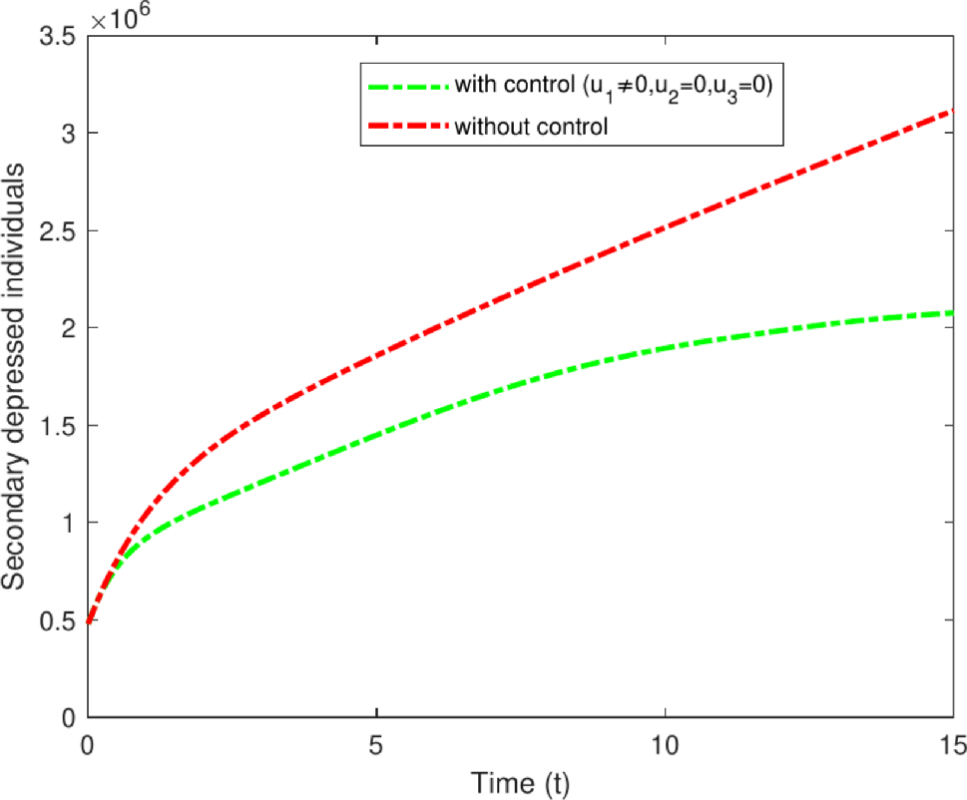
Variations of secondary depressed individuals with and without control for $u_1$ only.

The analysis explicitly investigates the optimal control parameter $u_1$, aimed at persons with primary-stage depression, and applies non-pharmacological interventions. This indicates that the incidence of individuals with secondary depression rapidly decreases, as those into the primary depressed compartment may not move to the secondary depressed compartment. Figure 13 illustrates the impact of $u_1$ control measures on patients with secondary depression, decreasing from 3,200,000 to 2,100,000 after 15 years.

#### Strategy B: $u_2$ only

**Fig. 14:**
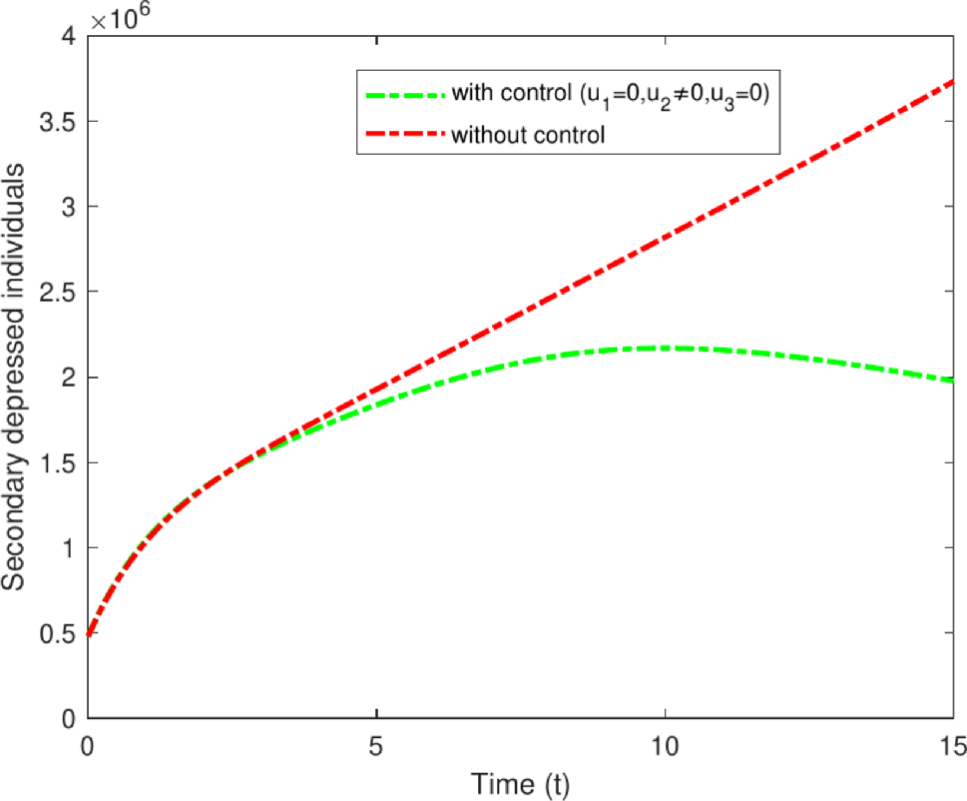
Variations of secondary depressed individuals with and without optimal control for $u_2$ only.

Strategy B involves the administration of pharmacological treatments for secondary-stage depression, including MAOIs, TCAs, SSRIs, and others. Although they can offer relief for those experiencing depression after a duration of two to three years, these treatments may also come with possible side effects, including dry mouth, trouble sleeping, blurred vision, fluctuations in weight, and memory issues^43^. This requires careful observation and oversight. Figure 14 clearly distinguishes between the scenarios with and without control in the study. The data indicates a decrease in secondary depressed cases from 3,200,000 to 1,950,000.

#### Strategy C: $u_3$ only

**Fig. 15:**
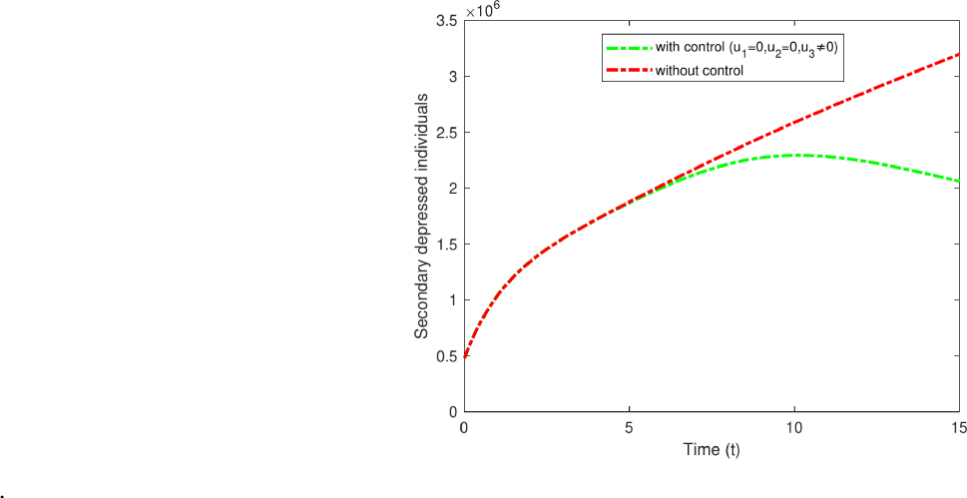
Variations of secondary depressed individuals with and without optimal control for $u_3$ only.

Strategy C entails offering improved treatment and therapy, which could encompass support for individuals receiving pharmacological interventions, including the use of tools like the PHQ-9 for assessing depression^44^. The effectiveness of these medications can vary from person to person, so it is important for healthcare providers to closely monitor their patient’s progress and adjust treatment as needed. Follow-up appointments: Depending on the case’s severity, follow-up appointments can be customized accordingly. For high-risk patients, follow-ups may be necessary every few days or weekly, whereas monthly check-ins could be adequate for those at lower risk. These methods typically result in improved recovery results. Only the control variable $u_3$ is utilized; the other two controls remain inactive. The findings illustrated in Fig. 15 highlight the effects of these control measures on individuals experiencing secondary depression. The results show a reduction in secondary depressed cases from 3,200,000 to 2,050,000, aiding in the prevention of relapse.

#### Strategy D: $u_1$ and $u_2$

**Fig. 16:**
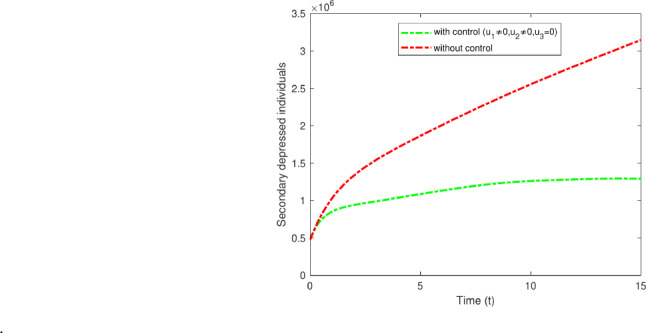
Variations of secondary depressed individuals, with and without optimal control for $u_1$ and $u_2$.

Strategy D illustrates the impact of applying two control measures, $u_1$ and $u_2$. The measures include the implementation of both pharmacological and non-pharmacological treatments for secondary depression, as well as providing respective treatments in both categories. Exercise ought to be considered a complementary approach alongside antidepressants instead of being viewed as an independent treatment. Combination therapy frequently results in increased levels of BDNF (brain-derived neurotrophic factor)^49^. This approach establishes the enhanced treatment and therapy control in primary and secondary depressed individual whereas the optimal control $u_3$ is zero. Figure 16 illustrate the impact of the optimal control measures $u_1$ and $u_2$ on individuals, leading to a decrease in secondary depression cases from 3,200,000 to 1,300,000.

#### Strategy E: $u_2$ and $u_3$

**Fig. 17:**
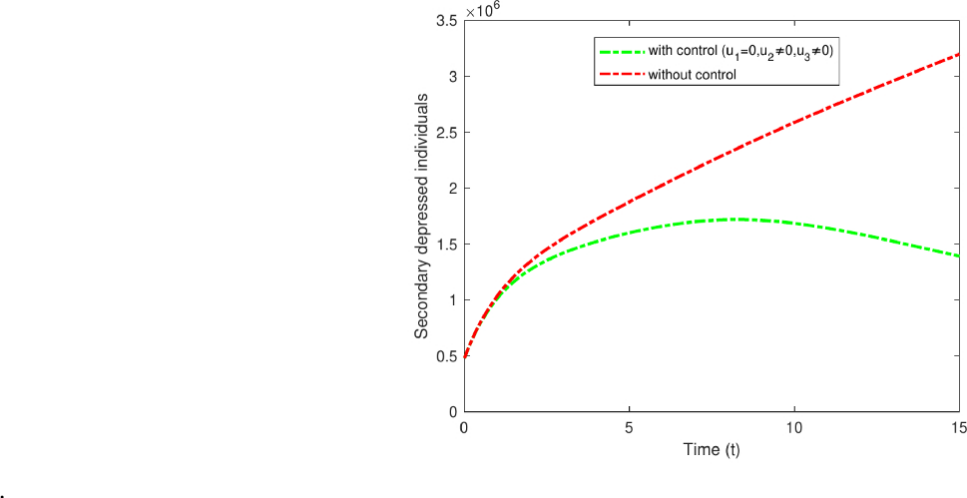
Variations of secondary depressed individuals with and without optimal control for $u_2$ and $u_3$.

Strategy E illustrates the combined impact of executing two simultaneous control measures, $u_2$ and $u_3$. These measures focus on recognizing individuals experiencing secondary depression and administering pharmacological interventions along with improved treatment approaches. The findings indicate that individuals undergoing cognitive therapy in conjunction with antidepressant medication treatment exhibit greater recovery rates from major depressive disorder than those receiving only antidepressant medication treatment^50^. By addressing both treatment methods at the same time, patients may see greater enhancements in their symptoms and overall well-being. Strategy E highlights the importance of investigating various treatment alternatives to enhance results for those experiencing major depressive disorder. The Figure17 illustrate the effects that decrease from 3,200,000 to 1,400,000 individuals experiencing secondary depression.

#### Strategy F: $u_1$ and $u_3$

**Fig. 18:**
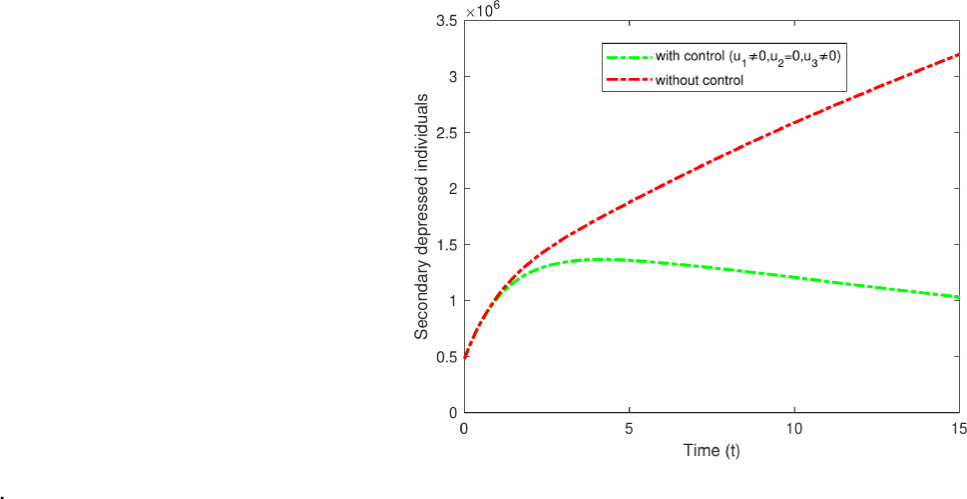
Variations of secondary depressed individuals with and without optimal control for all $u_1$ and $u_3$.

Strategy F entails the administration of pharmacological treatments $u_1$ in conjunction with improved treatment strategies $u_3$. This approach seeks to enhance the treatment’s efficacy by addressing both the symptoms and the root causes of the condition. Through the combination of pharmacological interventions and advanced strategies, patients could achieve enhanced outcomes and improved overall health. This approach helps a comprehensive personalized treatment strategy, catering to the individual needs of every patient. Moreover, integrating pharmacological treatments with improved strategies could potentially mitigate the risk of side effects linked to medication on its own. Overall, Strategy F provides a comprehensive method of treatment, enhancing long-term outcomes and quality of life for patients. Figure 18 illustrates the efforts that led to a reduction from 3,200,000 to 1,000,000 among individuals experiencing secondary depression.

#### Strategy G: All three optimal controls $u_1$, $u_2$ and $u_3$

**Fig. 19:**
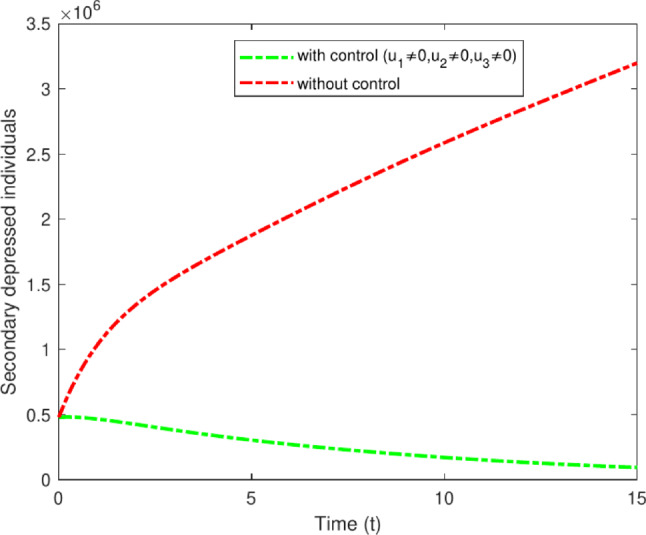
Variations of Secondary depressed individuals with and without optimal control for $u_1$, $u_2$ and $u_3$.

**Fig. 20:**
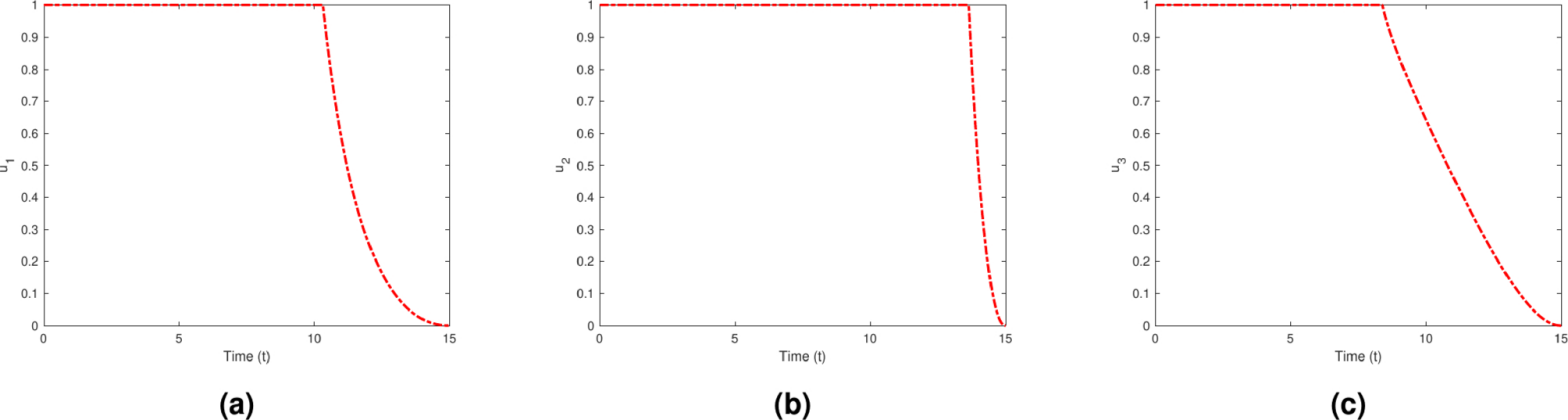
Control profile of (**a**) $u_1$, (**b**) $u_2$ and (**c**) $u_3$.

Strategy G represents the comprehensive execution of all control measures. The integration of various pharmacological and non-pharmacological treatments, along with the enhancement of treatment through regular monitoring, has resulted in a decrease in depression symptoms. Strategy G has effectively combined multiple treatment options to alleviate the symptoms of depression. Through the combination of various pharmacological and non-pharmacological strategies, individuals can obtain thorough care specific to their unique requirements. Furthermore, consistent observation and assessment of progress ensure the treatment strategy stays effective and yields enduring outcomes in enhancing mental well-being. The data illustrated in Fig. 19 indicates that strategy G is notably successful in controlling the population, decreasing the number of secondary depressed individuals from 3,200,000 to 200,000 over a period of 15 years. Figure 20 shows the control profiles of $u_1$ , $u_2$ and $u_3$. These figures indicate that the control profile $u_2$ should be sustained at 1 for an extended period compared to the other control. This control is closely linked with enhanced treatment strategies, which include providing quality counseling and allocating sufficient time to patients. Adherence to these measures by the entire population can significantly diminish the spread of the disease.

## Conclusion

In this study, we created and examined a mathematical model to understand the dynamics of depression in a community. The model takes into account both pharmacological and non-pharmacological treatments and distinguishes between primary and secondary depressed individuals. By incorporating the interaction of saboteur factors-those negatively influencing the mental health of others-the model offers a realistic and nuanced representation of depression dynamics. Through rigorous mathematical analysis, including the examination of four equilibrium points, we gained insights into the factors affecting depression persistence and prevalence. Stability analysis clarified the conditions under which depression can either become endemic or be eradicated from the population. Our data fitting and numerical simulations produced a realistic picture of depression trends, validated against actual data from Spain between 2011 and 2022. Sensitivity analysis identified parameters significantly influencing depression prevalence, providing potential targets for intervention. Contour plots demonstrated that as transmission rates ($\beta _2$), along with rates of transition from primary to secondary depression with and without saboteurs’ interaction ($\lambda _1$ and $\lambda _2$), decrease and treatment rates ($\psi _1$ and $\psi _2$) increase the basic reproduction number ($R_0$) declines. An extensive analysis of treatment effects, interactions with saboteurs, and other social influences confirmed that increases in transmission rates ($\beta _2$) result in higher numbers of depressed individuals. Conversely, higher recovery and treatment rates ($\gamma _1, \gamma _2, \psi _1, \psi _2$) correlate with fewer depressed individuals and an increase in recovered cases. Our analysis highlighted that the interaction with saboteurs substantially raises depression levels.

The proposed model includes optimal controls to determine the most effective combination of interventions. Strategies involving public awareness campaigns, pharmacological and non-pharmacological therapies, quality counseling, and limiting undesirable interactions were assessed. The model indicates that combining all three control measures is the most effective way to lower depression prevalence. Thus, early counseling and appropriate treatment can mitigate adverse effects, such as mental illness and suicide, significantly reducing depression rates. This emphasizes the critical importance of prompt and comprehensive interventions in enhancing mental health. In conclusion, this study presents a novel compartmental model of depression that categorizes individuals by mental health status while addressing the role of saboteurs in undermining community well-being. While most previous models have overlooked such influences, this work fills the gap by demonstrating their substantial impact on depression dynamics. However, limitations such as the assumption of homogeneous mixing remain, which may not fully capture the complexities of real-world social interactions. Future research can explore other social dynamics, such as positive influencers and community support networks, to better understand their effects on depression transmission. Additionally, incorporating a cost-effectiveness analysis can offer valuable insights for the practical implementation of control strategies.

## Supplementary Information

Supplementary Information.

## Data Availability

The datasets analyzed during the current study are available in the Statista repository at “https://www.statista.com/statistics/1173692/number-of-cases-of-depression-in-spain/”.
