# Supplementary material for: Mathematical modeling and optimal control of depression dynamics influenced by saboteurs
Source: Sci Rep. 2025 Feb 25;15:6773. doi: 10.1038/s41598-025-90357-w (PMC11861278; doi:10.1038/s41598-025-90357-w)
Supplement: Supplementary file 1 — Supplementary Information. [file 41598_2025_90357_MOESM1_ESM.pdf]

## Supplementary document

### Proof of the theorems in Stability analysis subsection

**Theorem 1.** *The equilibrium point* $E_0 = (\frac{\Lambda}{\mu}, 0, 0, 0, 0, 0, 0)$ *is locally asymptotically stable under some restriction on parameters.*

*Proof.* The Jacobain matrix of the given system at non-trivial point $E_0 = (\frac{\Lambda}{\mu}, 0, 0, 0, 0, 0, 0)$ is obtained as follows:

$$J_{E_0} = \begin{pmatrix} -\mu & -\beta_1 & 0 & -\beta_2 & 0 & 0 & 0 \\ 0 & \beta_1-\mu & 0 & 0 & 0 & 0 & 0 \\ 0 & 0 & -a_1 & \beta_2 & 0 & 0 & 0 \\ 0 & 0 & \lambda_2 & -a_2 & 0 & 0 & \omega \\ 0 & 0 & \psi_1 & 0 & -(\gamma_1+\mu) & 0 & 0 \\ 0 & 0 & 0 & \psi_2 & 0 & -(\mu+\gamma_2) & 0 \\ 0 & 0 & 0 & 0 & \gamma_1 & \gamma_2 & -(\omega+\mu) \end{pmatrix}$$

where

$$a_1 = (\lambda_2+\mu+\psi_1),\ a_2 = (\psi_2+\mu+\sigma)$$

The characteristic equation $|J_{E_0} - ZI| = 0$, we have the following two eigen values $-\mu$ and $\beta_1 - \mu$, where other five eigenvalues are the roots of the following equations

$$M^5 + C_1M^4 + C_2M^3 + C_3M^2 + C_4M + C_5$$

where,

$$\begin{aligned}
C_1 &= a_2+a_1+\gamma_2+\gamma_1+\omega+3\mu, \\
C_2 &= (\omega+\mu)(a_2+\mu+\gamma_2)+a_2(\mu+\gamma_2)+(a_2+\gamma_2+\omega+2\mu)(a_1+\gamma_1+\mu)+a_1(\gamma_1+\mu)-\lambda_2\beta_2, \\
C_3 &= a_1(\gamma_1+\mu)(a_2+\omega+\gamma_2+2\mu)+a_2(\mu+\gamma_2)(\omega+\mu)+(a_1+\gamma_1+\mu)[(\omega+\mu)(a_2+\mu+\gamma_2)+a_2(\mu+\gamma_2)] \\
&\quad -\psi_1\gamma_1\omega-\lambda_2\beta_2(\gamma_1+\gamma_2+3\mu), \\
C_4 &= a_2(\mu+\gamma_2)(\omega+\mu)(a_1+\gamma_1+\mu)+a_1(\gamma_1+\mu)(a_2+\mu+\gamma_2)-(a_1+\gamma_1+\mu)\gamma_1\psi_1\omega-\lambda_2\beta_2(\gamma_1+\mu)(\omega+\mu) \\
&\quad -(\gamma_1+\omega+2\mu)(\mu+\gamma_2)-\beta_2\psi_1\gamma_1\omega \\
C_5 &= a_1a_2(\mu+\gamma_2)(\omega+\mu)(\gamma_1+\mu)-\psi_1\gamma_1\omega a_1(\gamma_1+\mu)-\lambda_2\beta_2(\gamma_1+\mu)(\omega+\mu)(\mu+\gamma_2)-(\mu+\gamma_2)\beta_2\psi_1\gamma_1\omega
\end{aligned}$$

The equilibrium $E_0 = (\frac{\Lambda}{\mu}, 0, 0, 0, 0, 0, 0)$ is locally asymptotically stable is it satisfy the following Routh-Hurwitz criteria

$$\begin{aligned}
&C_i > 0,\ \text{for } i = 1,2,3,4,5 \\
&C_2C_1 - C_3^2 > 0, \\
&C_3(C_1C_2 - C_3^2) - C_4C_1^2 > 0, \\
&C_4(C_1C_3 - C_2^2) - C_5C_1^2 > 0, \\
&C_5(C_2C_3 - C_4^2)
\end{aligned}$$

. □

**Theorem 2.** *The depression free equilibrium point* $E_1 = (S^*, B^*, 0, 0, 0, 0, 0)$*, where* $S^* = \frac{\Lambda}{\beta_1}$ *and* $B^* = (\frac{\beta_1}{\mu} - 1)\frac{\Lambda}{\beta_1}$ *is locally asymptotically stable under some restriction on parameters.*

*Proof.* The jacobain matrix of the given system at non-trivial point $E_1 = (S^*, B^*, 0, 0, 0, 0, 0)$ is obtained by

$$J_{E_1} = \begin{pmatrix} -b_1-\mu & b_2 & b_3 & b_4 & b_3 & b_3 & b_3 \\ b_1 & -b_2-\mu & -b_3 & -b_3 & -b_3 & -b_3 & -b_3 \\ b_5 & 0 & b_6 & b_7 & 0 & 0 & 0 \\ 0 & 0 & b_8 & b_9 & 0 & 0 & \omega \\ 0 & 0 & \psi_1 & 0 & b_{10} & 0 & 0 \\ 0 & 0 & 0 & \psi_2 & 0 & b_{11} & 0 \\ 0 & 0 & 0 & 0 & \gamma_1 & \gamma_2 & -(\omega+\mu) \end{pmatrix}$$

where

$$b_1 = \frac{\beta_1 B^2}{(S+B)^2};\ b_2 = \frac{-\beta_1 S^2}{(S+B)^2},\ b_3 = \frac{\beta_1 BS}{(S+B)^2},\ b_4 = \frac{\beta_1 BS}{(S+B)^2} + \frac{\beta_2 S}{N},\ b_5 = \frac{\lambda_1 BP}{(S+B)^2} = 0,\ b_{11} = -(\mu+\gamma_2)$$

$$b_6 = -(\frac{\lambda_1 B}{(S+B)} + (\lambda_2+\mu+\psi_1),\ b_7 = \frac{-\beta_2 S}{(S+B)^2},\ b_8 = \frac{\lambda_1 B}{(S+B)} + \lambda_2,\ b_9 = -(\psi_2+\mu+\sigma),\ b_{10} = -(\gamma_1+\mu),$$

The characteristic equation $|J_{E_1} - YI| = 0$, and the eigenvalues are the roots of the following equation

$$Y^7 + G_1Y^6 + G_2Y^5 + G_3Y^4 + G_4Y^3 + G_5Y^2 + G_6Y + G_7 = 0$$

where

$$\begin{aligned}
G_1 &= b_1 + b_2 - b_6 - b_9 - b_{10} - b_{11} + 3\mu + \omega \\
G_2 &= b_6\,(b_9 - b_2 - b_1 + b_{10} + b_{11} - 3\mu - \omega) + b_9\,(b_6 - b_1 - b_2 + b_{10} + b_{11} - 3\mu - \omega) + b_{10}\,(b_9 + b_6 - b_1 - b_2 \\
&\quad + b_{11} - 3\mu - \omega) + b_{11}\,(b_9 + b_6 - b_1 - b_2 + b_{10} - 3\mu - \omega) + b_3b_5 + b_7b_8 + 2b_1\mu + 2b_2\mu + b_1\omega + b_2\omega + 2\mu\omega + 3\mu^2 \\
G_3 &= (b_1 + b_2 - 3b_6 - 3b_9 - 3b_{10} - 3b_{11})\mu^2 + \mu^2\omega + \mu^3 + (b_1b_6b_9 + b_1b_6b_{10} + b_1b_6b_{11} + b_2b_6b_9 + b_2b_6b_{10} + b_2b_6b_{11} \\
&\quad + b_1b_9b_{10} + b_1b_9b_{11} + b_2b_9b_{10} + b_2b_9b_{11} + b_1b_{10}b_{11} + b_2b_{10}b_{11}) - (b_1b_7b_8 + b_2b_7b_8 + b_6b_9b_{10} + b_6b_9b_{11} \\
&\quad + b_6b_{10}b_{11} + b_9b_{10}b_{11}) + b_7b_8b_{10} + b_7b_8b_{11} + (b_3b_5b_9 + b_3b_5b_{10} + b_3b_5b_{11} - b_4b_5b_8) - b_3b_5\psi_1 - 2(b_1b_6 \\
&\quad + b_2b_6 + b_3b_5 + b_1b_9 + b_1b_{10} + b_2b_9 + b_1b_{11} + b_2b_{10} + b_2b_{11})\mu + 3(b_6b_9 + b_6b_{10} + b_6b_{11} + b_9b_{10} + b_9b_{11} + b_{10}b_{11})\mu \\
&\quad - (b_1b_6 + b_2b_6 + b_3b_5 + b_1b_9 + b_1b_{10} + b_2b_9 + b_1b_{11} + b_2b_{10} + b_2b_{11})\omega + (b_6b_9 + b_6b_{10} + b_6b_{11} + b_9b_{10} \\
&\quad + b_9b_{11} + b_{10}b_{11})\omega + b_1\mu\omega + b_2\mu\omega - 2(b_6 + b_9 + b_{10} + b_{11})\mu\omega - \gamma_2\omega\psi_2 \\
G_4 &= -(b_9 + b_{10} + b_{11} + b_6)\,\mu^3 + (3b_6b_9 - b_1b_6 - b_2b_6 - b_3b_5 - b_1b_9 - b_1b_{10} - b_2b_9 - b_1b_{11} - b_2b_{10} - b_2b_{11} \\
&\quad - 3b_7b_8 + 3b_6b_{10} + 3b_6b_{11} + 3b_9b_{10} + 3b_9b_{11} + 3b_{10}b_{11} - (b_6 + b_9 + b_{10} + b_{11})\omega)\,\mu^2 + (-b_1b_6 - b_2b_6 - b_3b_5 \\
&\quad + -b_1b_9 - b_1b_{10} - b_2b_9 - b_1b_{11} - b_2b_{10} - b_2b_{11} + 2b_6b_9 + 2b_6b_{10} + 2b_6b_{11} + 2b_9b_{10} + 2b_9b_{11} + 2b_{10}b_{11})\,\mu\omega \\
&\quad + (b_2b_3b_5b_8 - b_2b_4b_5b_8) + 2\,(b_1b_6b_9 + b_1b_6b_{10} + b_1b_6b_{11} + b_2b_6b_9 + b_2b_6b_{10} + b_2b_6b_{11} \\
&\quad + b_3b_5b_9 + b_3b_5b_{10} + b_3b_5b_{11} + b_1b_9b_{10} + b_1b_9b_{11} + b_2b_9b_{10} + b_2b_9b_{11} + b_2b_{10}b_{11})\mu \\
&\quad - 3\,(b_6b_9b_{10} + b_6b_9b_{11} + b_6b_{10}b_{11} + b_9b_{10}b_{11})\,\mu \\
G_5 &= 3\,b_6\,b_9\,\mu^2 - b_9\,\mu^3 - b_{10}\,\mu^3 - b_{11}\,\mu^3 - b_1\,b_6\,\mu^2 - b_2\,b_6\,\mu^2 - b_3\,b_5\,\mu^2 - b_1\,b_9\,\mu^2 - b_1\,b_{10}\,\mu^2 - b_2\,b_9\,\mu^2 \\
&\quad - b_1\,b_{11}\,\mu^2 - b_2\,b_{10}\,\mu^2 - b_2\,b_{11}\,\mu^2 - b_6\,\mu^3 - 3\,b_7\,b_8\,\mu^2 + 3\,b_6\,b_{10}\,\mu^2 + 3\,b_6\,b_{11}\,\mu^2 + 3\,b_9\,b_{10}\,\mu^2 + 3\,b_9\,b_{11}\,\mu^2 \\
&\quad + 3\,b_{10}\,b_{11}\,\mu^2 - 2\,b_3\,b_5\,\mu\,\psi_1 + b_2\,b_3\,b_5\,b_8 - b_2\,b_4\,b_5\,b_8 - b_1\,b_6\,b_9\,b_{10} + b_1\,b_7\,b_8\,b_{10} - b_1\,b_6\,b_9\,b_{11} + b_1\,b_7\,b_8\,b_{11} \\
&\quad - b_2\,b_6\,b_9\,b_{10} + b_2\,b_7\,b_8\,b_{10} - b_3\,b_5\,b_9\,b_{10} + b_4\,b_5\,b_8\,b_{10} - b_1\,b_6\,b_{10}\,b_{11} - b_2\,b_6\,b_9\,b_{11} + b_2\,b_7\,b_8\,b_{11} - b_3\,b_5\,b_9\,b_{11} \\
&\quad + b_4\,b_5\,b_8\,b_{11} - b_2\,b_6\,b_{10}\,b_{11} - b_3\,b_5\,b_{10}\,b_{11} - b_1\,b_9\,b_{10}\,b_{11} - b_2\,b_9\,b_{10}\,b_{11} + b_6\,b_9\,b_{10}\,b_{11} - b_7\,b_8\,b_{10}\,b_{11} \\
&\quad + 2\,b_1\,b_6\,b_9\,\mu - 2\,b_1\,b_7\,b_8\,\mu + 2\,b_1\,b_6\,b_{10}\,\mu + 2\,b_2\,b_6\,b_9\,\mu - 2\,b_2\,b_7\,b_8\,\mu + 2\,b_3\,b_5\,b_9\,\mu - 2\,b_4\,b_5\,b_8\,\mu \\
&\quad + 2\,b_1\,b_6\,b_{11}\,\mu + 2\,b_2\,b_6\,b_{10}\,\mu + 2\,b_3\,b_5\,b_{10}\,\mu + 2\,b_2\,b_6\,b_{11}\,\mu + 2\,b_3\,b_5\,b_{11}\,\mu + 2\,b_1\,b_9\,b_{10}\,\mu + 2\,b_1\,b_9\,b_{11}\,\mu \\
&\quad + 2\,b_2\,b_9\,b_{10}\,\mu + 2\,b_1\,b_{10}\,b_{11}\,\mu + 2\,b_2\,b_9\,b_{11}\,\mu + 2\,b_2\,b_{10}\,b_{11}\,\mu - 3\,b_6\,b_9\,b_{10}\,\mu + 3\,b_7\,b_8\,b_{10}\,\mu - 3\,b_6\,b_9\,b_{11}\,\mu \\
&\quad + 3\,b_7\,b_8\,b_{11}\,\mu - 3\,b_6\,b_{10}\,b_{11}\,\mu - 3\,b_9\,b_{10}\,b_{11}\,\mu - b_3\,b_5\,b_8\,\psi_2 + b_3\,b_5\,b_9\,\psi_1 + b_3\,b_5\,b_{11}\,\psi_1 - b_3\,b_5\,\gamma_1\,\psi_1
\end{aligned}$$

$$
\begin{aligned}
G_6 \;=\;& \mu^3 \left(b_7 b_8 (b_{10}+b_{11}) - b_6 b_9 (b_{10}+b_{11}) - b_6 b_{10} b_{11} - b_9 b_{10} b_{11}\right) + \mu^2 \left(b_1 \left(b_7 b_8 (b_{10}+b_{11}) - b_6 b_9 (b_{10}+b_{11})\right.\right.\\
& \left. -b_6 b_{10} b_{11} - b_9 b_{10} b_{11}\right) + b_2 \left(b_7 b_8 (b_{10}+b_{11}) - b_6 b_9 (b_{10}+b_{11}) - b_6 b_{10} b_{11} - b_9 b_{10} b_{11}\right) + b_3 b_5 \left(b_9(\psi_1 + b_{10}\right.\\
& \left. +b_{11}) - b_8 \psi_2 - b_{10} b_{11}\right) + b_4 b_5 \left(b_8 (b_{10}+b_{11}) - b_9 b_{10} b_{11}\right) + 3 b_6 b_9 b_{10} b_{11} - 3 b_7 b_8 b_{10} b_{11} - b_3 b_5 b_8 \psi_2\\
& \left. +b_3 b_5 b_9 \psi_1 + b_3 b_5 b_{11} \psi_1\right) + \mu \left(b_2 \left(2 b_6 b_9 b_{10} b_{11} - 2 b_7 b_8 b_{10} b_{11} - b_3 b_5 (b_8 (1+\psi_2) - b_9 (1+\psi_1) - b_{11} (1+\psi_1)\right)\right)\\
& +b_4 b_5 \left(2 b_8 b_{10} b_{11} - b_8 (b_{10}+b_{11})\right)) + 2 b_1 \left(b_6 b_9 b_{10} b_{11} - b_7 b_8 b_{10} b_{11}\right) + b_3 b_5 \left(2 b_8 b_{10} \psi_2 - 2 b_9 b_{11} \psi_1 + b_9 \gamma_1 \psi_1\right.\\
& \left. -b_8 \gamma_2 \psi_2 + b_{11} \gamma_1 \psi_1\right) - b_4 b_5 \left(2 b_8 b_{10} b_{11}\right)) + b_2 b_3 b_5 \left(b_8 b_{10} b_{11} - b_8 (b_{10}+b_{11})\right)) - b_2 b_4 b_5 (b_8 (b_{10}+b_{11}))\\
G_7 \;=\;& (b_6 b_9 b_{10} b_{11} - b_7 b_8 b_{10} b_{11}) \mu^3 + [(b_1 b_6 b_9 b_{10} b_{11} - b_1 b_7 b_8 b_{10} b_{11} + b_2 b_6 b_9 b_{10} b_{11} - b_2 b_7 b_8 b_{10} b_{11}\\
& +b_3 b_5 b_9 b_{10} b_{11} - b_4 b_5 b_8 b_{10} b_{11} + b_6 b_9 b_{10} b_{11} \omega - b_7 b_8 b_{10} b_{11} \omega + b_3 b_5 b_8 b_{10} \psi_2 - b_3 b_5 b_9 b_{11} \psi_1 - b_6 b_{10} \gamma_2 \omega \psi_2\\
& +b_7 b_{11} \gamma_1 \omega \psi_1] \mu^2 + [(b_2 b_3 b_5 b_8 b_{10} b_{11} - b_2 b_4 b_5 b_8 b_{10} b_{11} + b_2 b_3 b_5 b_8 b_{10} \omega - b_2 b_4 b_5 b_8 b_{10} \omega + b_1 b_6 b_9 b_{10} b_{11} \omega\\
& -b_1 b_7 b_8 b_{10} b_{11} \omega + b_2 b_6 b_9 b_{10} b_{11} \omega - b_2 b_7 b_8 b_{10} b_{11} \omega + b_3 b_5 b_9 b_{10} b_{11} \omega - b_4 b_5 b_8 b_{10} b_{11} \omega + b_3 b_5 b_8 b_{10} \gamma_2 \psi_2\\
& -b_3 b_5 b_9 b_{11} \gamma_1 - b_2 b_3 b_5 b_{11} \gamma_1 \omega \psi_1 + b_2 b_4 b_5 b_{11} \gamma_1 \omega \psi_1 + b_3 b_5 b_8 b_{10} \mu \omega \psi_2 - b_3 b_5 b_9 b_{11} \mu \omega \psi_1 - b_1 b_6 b_{10} \gamma_2 \mu \omega \psi_2\\
& +b_1 b_7 b_{11} \gamma_1 \mu \omega \psi_1 - b_2 b_6 b_{10} \gamma_2 \mu \omega \psi_2 + b_2 b_7 b_{11} \gamma_1 \mu \omega \psi_1 - b_3 b_5 b_{10} \gamma_2 \mu \omega \psi_2 + b_4 b_5 b_{11} \gamma_1 \mu \omega \psi_1 - b_3 b_5 \gamma_1 \mu \omega \psi_1 \psi_2\\
& +b_3 b_5 \gamma_2 \mu \omega \psi_1 \psi_2) \mu
\end{aligned}
$$

If the following Routh-Hurwitz criteria satisfied, then $E_1$ will be locally asymptotically stable.

$$
\begin{aligned}
& -G_i > 0, \text{ for } i = 1,2,3,4,5,6,7\\
& G_7 G_6 G_5 - G_5^2 > 0\\
& (G_7 G_6 G_5 - G_5^2)(G_7 G_4 G_6 - G_6^2 - G_7^2 G_3) - G_6 G_5 (G_7 G_3 G_5 - G_4^2) > 0\\
& G_7 \left(G_6 \left(G_5 (G_4 G_3 - G_2 G_5) - G_5 (G_3 G_2 - G_1 G_4)\right) + G_6^2 G_2\right) - G_5 G_6 G_3^2 + G_6 G_5 G_2 G_3 > 0\\
& G_7 G_6 G_5 G_4 G_3 G_2 - G_5^2 G_6 G_4 G_3 - G_6 G_5 G_3^2 + G_6 G_5 G_4 G_2 - G_6 G_5 G_1 G_4 G_3 + G_6 G_4^2 G_2 > 0
\end{aligned}
$$

□

**Theorem 3.** *The saboteurs population free equilibrium point $E_2 = (S^*, 0, P^*, D^*, M^*, C^*, R^*)$, is locally asymptotically stable under some restriction on parameters.*

*Proof.* The Jacobain matrix of the given system at non-trivial point $E_2 = (S^*, 0, P^*, D^*, M^*, C^*, R^*)$, is obtained as follows:

$$
J_{E_2} = \begin{pmatrix}
-(a_1+\mu) & a_2 & a_3 & a_4 & a_3 & a_3 & a_3\\
0 & a_5 & 0 & 0 & 0 & 0 & 0\\
a_1 & a_6 & -(a_3+\mu) & -a_4 & -a_3 & -a_3 & -a_3\\
0 & a_7 & \lambda_2 & a_8 & 0 & 0 & \omega\\
0 & 0 & \psi_1 & 0 & -(\gamma_1+\mu) & 0 & 0\\
0 & 0 & 0 & \psi_2 & 0 & -(\mu+\gamma_2) & 0\\
0 & 0 & 0 & 0 & \gamma_1 & \gamma_2 & -(\omega+\mu)
\end{pmatrix}
$$

where

$$
N = (S+P+D+M+C+R),\; a_1 = \frac{\beta_2 DN - \beta_2 DS}{N^2},\; a_2 = \frac{-\beta_1 S}{N} + \frac{\beta_2 DS}{N^2},\; a_4 = -\left(\frac{\beta_2 SN - \beta_2 DS}{N^2}\right)
$$

$$
a_3 = \frac{\beta_2 DS}{N^2},\; a_5 = \frac{\beta_1 S}{N},\; a_6 = -\left(\frac{\beta_2 DS}{N^2} - \frac{\lambda_1 P}{N}\right),\; a_7 = \frac{\lambda_1 P}{N},\; a_8 = -\left(\frac{\beta_2 DS}{N^2} - \frac{-\lambda_1 P}{N}\right)
$$

The characteristic equation $|J_{E_2} - ZI| = 0$, we have the following eigen value
$a_5 = \frac{\beta_1 S}{N}$
And the other eigen values are obtained from the following equation

$$
Z^6 + H_1 Z^5 + H_2 Z^4 + H_3 Z^3 + H_4 Z^2 + H_5 Z + H_6 = 0
$$

where

$$
\begin{aligned}
H_1 &= a_1+a_3-a_8+\gamma_1+\gamma_2+5\mu+\omega \\
H_2 &= a_1(\gamma_1+\gamma_2+4\mu+\omega)+a_3(\gamma_1+\gamma_2+4\mu+\omega+\psi_1)-(a_3+a_1+a_8)(\gamma_1+\gamma_2+\omega)-a_1a_8-a_4\lambda_2+\gamma_1\gamma_2+10\mu^2 \\
&\quad +4\mu\omega+4(\gamma_1+\gamma_2)\mu \\
H_3 &= (6a_1+6a_3-10a_8+6\gamma_1+6\gamma_2+6\omega)\mu^2+10\mu^3-(a_1+a_3)a_8(\gamma_1+\gamma_2)-a_3a_8\psi_1+(a_4\gamma_1+a_4\gamma_2+4a_4\lambda_2+3a_3\lambda_2)\mu \\
&\quad +(a_4\lambda_2+3a_1+3a_3)\omega+(3\gamma_1+3\gamma_2+3a_1+3a_3)\mu\omega+(1+a_3)\gamma_1\psi_1+(1+a_3)\gamma_2\psi_1+\gamma_1\gamma_2(\mu+\omega)-\gamma_2\omega\psi_2 \\
H_4 &= \mu^4(5+4\omega)+\mu^3(4a_1+4a_3-10a_8+4\gamma_1+4\gamma_2)+\mu^2(3\gamma_1\omega+3\gamma_2\omega-6a_1a_8-6a_3a_8+3a_1\gamma_1+3a_1\gamma_2+3a_3\gamma_1+3a_3\gamma_2) \\
&\quad +\mu^2(6a_4\lambda_2+3\gamma_1\gamma_2-6a_8\gamma_1-6a_8\gamma_2-6a_8\omega+3a_3\psi_1)+\mu(3a_4\gamma_1\lambda_2+3a_4\gamma_2\lambda_2+2a_1\gamma_1\omega+2a_1\gamma_2\omega+2a_3\gamma_1\omega \\
&\quad +2a_3\gamma_2\omega-3a_8\gamma_1\omega-3a_8\gamma_2\omega)+(2a_3\gamma_1\mu+2a_3\gamma_2\mu-a_1\gamma_2\omega\psi_2+a_4\gamma_1\omega\psi_1+a_3\gamma_2\omega\psi_1+a_4\gamma_2\lambda_2\omega-a_3\gamma_2\omega\psi_2) \\
&\quad +(3a_4\lambda_2\mu\omega+3a_3\lambda_2\mu\psi_2+2\gamma_1\gamma_2\mu\omega+a_3\lambda_2\omega\psi_2+2a_3\mu\omega\psi_1-\gamma_1\gamma_2\omega\psi_2-3\gamma_2\mu\omega\psi_2) \\
&\quad -(a_1a_8\gamma_1\gamma_2+a_3a_8\gamma_1\gamma_2+3a_1a_8\gamma_1\mu+3a_1a_8\gamma_2\mu+3a_3a_8\gamma_1\mu+3a_3a_8\gamma_2\mu+a_1a_8\gamma_1\omega+a_1a_8\gamma_2\omega+a_3a_8\gamma_1\omega+a_3a_8\gamma_2\omega) \\
&\quad +(a_4\gamma_1\gamma_2\lambda_2+2a_1\gamma_1\gamma_2\mu+2a_3\gamma_1\gamma_2\mu-3a_8\gamma_1\gamma_2\mu-3a_1a_8\mu\omega-3a_3a_8\mu\omega+a_1\gamma_1\gamma_2\omega+a_3\gamma_1\gamma_2\omega-a_8\gamma_1\gamma_2\omega-3a_3a_8\mu\psi_1) \\
H_5 &= \mu^5+(a_1+a_3-5a_8+\gamma_1+\gamma_2+\omega)\mu^4+[\gamma_1\omega+\gamma_2\omega+a_1\gamma_1+a_1\gamma_2+a_3\gamma_1+a_3\gamma_2-4a_1a_8-4a_3a_8+4a_4\lambda_2 \\
&\quad -4a_8(\gamma_1+\gamma_2)+a_1\omega+a_3\omega-4a_8\omega+a_3\psi_1]\mu^3+[a_1\gamma_1\gamma_2+a_3\gamma_1\gamma_2-3(a_1a_8\gamma_1+a_1a_8\gamma_2+a_3a_8\gamma_1+a_3a_8\gamma_2) \\
&\quad +3a_4\lambda_2(\gamma_1+\gamma_2)-3(a_1a_8+a_3a_8)(\gamma_1+\gamma_2)]\mu^2+[-2(a_1a_8+a_3a_8)\gamma_1\gamma_2+(a_1\gamma_1\gamma_2+a_3\gamma_1\gamma_2-2a_8\gamma_1\gamma_2) \\
&\quad +2a_3\gamma_1\lambda_2\psi_2+2a_3\gamma_2\lambda_2\psi_2+a_4\gamma_1\gamma_2\lambda_2\omega-a_3a_8\gamma_2\omega\psi_1]\mu+[-2\gamma_1\gamma_2+2a_3\lambda_2\psi_2] \\
H_6 &= -a_8\mu^5+(a_4\lambda_2-a_1a_8-a_3a_8-a_8\gamma_1-a_8\gamma_2-a_8\omega)\mu^4+(-a_1a_8\gamma_1-a_1a_8\gamma_2-a_3a_8\gamma_1-a_3a_8\gamma_2-a_8\gamma_1\gamma_2-a_1a_8\omega \\
&\quad -a_3a_8\omega-a_3a_8\psi_1+a_4\gamma_1\lambda_2+a_4\gamma_2\lambda_2+a_4\lambda_2\omega+a_3\lambda_2\psi_2-\gamma_2\omega\psi_2)\mu^3+(-a_1a_8\gamma_1\gamma_2-a_3a_8\gamma_1\gamma_2-a_1a_8\gamma_1\omega \\
&\quad -a_1a_8\gamma_2\omega-a_3a_8\gamma_1\omega-a_3a_8\gamma_2\omega-a_3a_8\gamma_1\psi_1-a_3a_8\gamma_2\psi_1+a_4\gamma_1\gamma_2\lambda_2-a_8\gamma_1\gamma_2\omega-a_3\mu^2\omega\psi_1+a_4\gamma_1\lambda_2\omega \\
&\quad +a_4\gamma_2\lambda_2\omega+a_3\gamma_1\lambda_2\psi_2+a_3\gamma_2\lambda_2\psi_2-a_1\gamma_2\omega\psi_2+a_4\gamma_1\omega\psi_1-a_3\gamma_2\omega\psi_2+a_3\lambda_2\omega\psi_2-\gamma_1\gamma_2\omega\psi_2)\mu^2 \\
&\quad +(-a_1a_8\gamma_1\gamma_2\omega-a_3a_8\gamma_1\gamma_2\omega-a_3a_8\gamma_1\gamma_2\psi_1-a_3a_8\gamma_2\omega\psi_1+a_4\gamma_1\gamma_2\lambda_2\omega+a_3\gamma_1\gamma_2\lambda_2\psi_2-a_1\gamma_1\gamma_2\omega\psi_2-a_3\gamma_1\gamma_2\omega\psi_2 \\
&\quad +a_4\gamma_1\gamma_2\omega\psi_1+a_3\gamma_1\lambda_2\omega\psi_2+a_3\gamma_1\omega\psi_1\psi_2-a_3\gamma_2\omega\psi_1\psi_2)\mu
\end{aligned}
$$

If the following conditions hold, $E_2$ will be locally asymptotically stable according to the Routh-Hurwitz criteria:

$$
\begin{aligned}
-H_i &> 0 \text{ for } i=1,2,3,4,5,6 \\
-(H_1H_2H_3) &> H_3^2+H_1^2H_4 \\
-(H_1H_4-H_5)(H_1H_2H_3-H_3^2-H_1^2H_4) &> H_5(H_1H_2-H_3)^2H_1H_5^2>0 \\
-(H_1H_5^2-H_2H_6)(H_1H_2H_3-H_3^2-H_1^2H_4) &> H_6(H_1H_2-H_3)^2H_1H_6^2>0
\end{aligned}
$$

□

**Theorem 4.** *The Endemic equilibrium point $E_3=(S^{**},B^{**},P^{**},D^{**},M^{**},C^{**},R^{**})$, is locally asymptotically stable under some restriction on parameters.*

*Proof.* The Jacobain matrix of the given system at non-trivial point $E_3=(S^{**},B^{**},P^{**},D^{**},M^{**},C^{**},R^{**})$ is obtained as follows:

$$
J_{E_3} = \begin{pmatrix}
a_1 & a_2 & a_3 & a_4 & a_3 & a_3 & a_3 \\
a_5 & a_6 & a_7 & a_7 & a_7 & a_7 & a_7 \\
a_8 & a_9 & a_{10} & a_{11} & a_{12} & a_{12} & a_{12} \\
a_{15} & a_{14} & a_{15} & a_{16} & a_{13} & a_{13} & a_{13}+\omega \\
0 & 0 & \psi_1 & 0 & a_{17} & 0 & 0 \\
0 & 0 & 0 & \psi_2 & 0 & a_{18} & 0 \\
0 & 0 & 0 & 0 & \gamma_1 & \gamma_2 & -(\mu+\omega)
\end{pmatrix}
$$

where

$$
a_1=-\left(\frac{(\beta_1BN-\beta_1BS)}{N^2}+\frac{(\beta_2DN-\beta_2DS)}{N^2}+\mu\right),\ a_2=-\frac{(\beta_1SN-\beta_1BS)}{N^2}+\frac{\beta_2DS}{N^2},\ a_{17}=-(\gamma_1+\mu),
$$

$$
a_3=\frac{(\beta_1BS=\beta_2DS)}{N^2},\ a_4=\frac{\beta_1BS}{N^2}-\frac{(\beta_2SN-\beta_2DS)}{N^2},\ a_5=\frac{\beta_1BN-\beta_1BS}{N^2},\ a_7=-\frac{\beta_1BS}{N^2},\ a_{18}=-(\gamma_2+\mu),
$$

$$a_6 = \frac{(\beta_1 SN - \beta_1 BS)}{N^2} - \mu,\ a_8 = \frac{(\beta_2 DN - \beta - 2DS)}{N^2} + \frac{\lambda_1 BP}{N^2},\ a_9 = \frac{(\lambda_1 PN - \lambda_1 BP)}{N^2} - \frac{\beta_2 DS}{N^2},$$
$$a_{10} = \frac{(\lambda_1 BN - \lambda_1 BP)}{N^2} - \frac{\beta_2 DS}{N^2} - (\lambda_2 + \mu + \psi_1),\ a_{11} = \frac{(\beta_2 SN - \beta_2 DS)}{N^2} + \frac{\lambda_1 BP}{N^2}, a_{13} = \frac{-\lambda_1 BP}{N^2},$$
$$a_{12} = \frac{(\lambda_1 BP - \beta_2 DS)}{N^2},\ a_{14} = \frac{(\lambda_1 PN - \lambda_1 BP)}{N^2},\ a_{15} = \frac{(\lambda_1 BN - \lambda_1 BP)}{N^2} + \lambda_2,\ a_{16} = -(\psi_2 + \mu + \sigma) - \frac{\lambda_1 BP}{N^2}.$$

The characteristic equation $|J_{E_3} - XI| = 0$ and the eigen values are the roots of the following equations,

$$X^7 + K_1 X^6 + K_2 X^5 + K_3 X^4 + K_4 X^3 + K_5 X^2 + K_6 X + K_7 = 0.$$

The coefficient $K_i'S$ can be found use any software with symbolic computation. If the following Routh-Hurwitx criteria holds, then the equilibrium point$E_3$ is locally asymptotically stable;

$$\begin{aligned}
&-K_i > 0,\ \text{for } i = 1,2,3,4,5,6,7\\
&K_7 K_6 K_5 - K_5^2 > 0,\\
&(K_7 K_6 K_5 - K_5^2)(K_7 K_4 K_6 - K_6^2 - K_7^2 K_3) - K_6 K_5 (K_7 K_3 K_5 - K_4^2) > 0,\\
&K_7 \left(K_6 \left(K_5 (K_4 K_3 - K_2 K_5) - K_5 (K_3 K_2 - K_1 K_4)\right) + K_6^2 K_2\right) - K_5 K_6 K_3^2 + K_6 K_5 K_2 K_3 > 0,\\
&K_7 K_6 K_5 K_4 K_3 K_2 - K_5^2 K_6 K_4 K_3 - K_6 K_5 K_3^2 + K_6 K_5 K_4 K_2 - K_6 K_5 K_1 K_4 K_3 + K_6 K_4^2 K_2 > 0.
\end{aligned}$$

□
